# Strategies for mitigating artificial intelligence bias in healthcare: a systematic review

**DOI:** 10.1093/jamiaopen/ooag081

**Published:** 2026-06-03

**Authors:** Kais Gadhoumi, Rashaud Senior, Sijia Wei, Leila Ledbetter, Michael D Green, Bethany D Bonner, Yvonne Mosley, Katie Seidler, Kristle Green, Donghwan Lee, Chuan Hong, Vincent Guilamo Ramos, Michael P Cary

**Affiliations:** Duke University School of Nursing, Durham, NC, 27710, United States; Avance Care, Raleigh, NC, United States; Duke University School of Medicine, Durham, NC, 27710, United States; Feinberg School of Medicine and Kellogg School of Management, Northwestern University, Chicago, IL, United States; Evidence Synthesis Information Scientist at the Virginia Tech Blacksburg, VA, 24061, United States; Duke University School of Medicine, Durham, NC, 27710, United States; Duke University School of Medicine, Durham, NC, 27710, United States; Triad HealthCare Network, Greensboro, NC, 27401, United States; Belk College of Business, University of North Carolina at Charlotte, Charlotte, NC, 28223, United States; Duke University School of Medicine, Durham, NC, 27710, United States; Food and Drug Administration, Silver Spring, MD, 20993, United States; Food and Drug Administration, Silver Spring, MD, 20993, United States; Duke University School of Nursing, Durham, NC, 27710, United States; Duke University School of Medicine, Durham, NC, 27710, United States; Johns Hopkins School of Nursing, Baltimore, MD, 20001, United States; Duke University School of Nursing, Durham, NC, 27710, United States; Duke University School of Medicine, Durham, NC, 27710, United States

**Keywords:** artificial intelligence, algorithms, delivery of healthcare, socioeconomic factors

## Abstract

**Objectives:**

Artificial intelligence is used in healthcare to identify and manage health conditions across diverse patient populations and clinical settings, but biases in algorithms can perpetuate and exacerbate inequalities in health and healthcare delivery. While some research has focused on addressing this critical issue, there is a lack of comprehensive information on the types of strategies employed for bias mitigation in the use of artificial intelligence in healthcare and the effectiveness of these strategies. The objective of this review was to address this lack of information by identifying, categorizing, and describing the effectiveness of bias reduction strategies and fairness metrics in healthcare algorithms.

**Materials and Methods:**

Following the Preferred Reporting Items for Systematic reviews and Meta-Analyses guidelines, this study categorizes and evaluates the effectiveness of bias reduction strategies and fairness metrics identified in a previous scoping review.

**Results:**

The review included 35 studies. The findings are related to the stages of the algorithm lifecycle and are aligned with the authors’ institutional governance structure for algorithm development, silent evaluation, effectiveness evaluation, and deployment. The majority (60%) of the identified strategies were implemented during the algorithm development and silent evaluation stages, and all studies utilized group fairness metrics for performance measurement. Most studies (85%) reported effective bias reduction, while only a few reported ineffectiveness (8%) or no effect (5%).

**Conclusion:**

There is a significant opportunity for model developers and end users to identify and reduce bias, particularly during model design. When evaluating strategy effectiveness, efforts should be measured using evidenced-based fairness metrics—such as group-based metrics—to ensure effectiveness and interpretability.

## Background and significance

Algorithms play a pivotal role in identifying and managing health conditions across diverse patient populations and clinical settings. However, research has highlighted the presence of biases in algorithms, leading to downstream issues such as differential disease detection or care provision among patient populations, often unbeknownst to users.^1–11^ These users—encompassing researchers, data scientists, and clinicians—must be able to identify and reduce these biases because bias detection and mitigation are essential to ensuring equitable and trustworthy artificial intelligence (AI) systems in healthcare.^12–14^ It is imperative to identify and address biases that may be inadvertently incorporated into algorithms because such biases can perpetuate and exacerbate existing inequalities in health and healthcare delivery. When algorithms inherit biases from their training data or design, they risk producing discriminatory outcomes, such as unequal access to care, misdiagnoses, or suboptimal treatment recommendations for marginalized groups. Moreover, these biases can undermine trust in AI technologies, create ethical and legal challenges, and ultimately hinder the equitable advancement of healthcare. Addressing these biases is not only a technical requirement but also a moral obligation to ensure AI systems promote fairness, improve patient outcomes across diverse populations, and contribute to reducing longstanding disparities in healthcare.

Given the profound potential of bias in AI to influence health outcomes, there have been efforts to develop strategies to identify and address such biases. For example, Section 1557 of the Patient Protection and Affordable Care Act issued in August 2022 prohibits discrimination on the basis of race, color, national origin, sex, age, or disability in healthcare.^15^ Since its issuance, the United States Department of Health and Human Services has emphasized the need to address discrimination and potential bias in clinical algorithms.

Despite the contributions on bias mitigation in healthcare AI in the literature, there remain 2 notable gaps. First, there is a lack of comprehensive information on the types of strategies employed for bias mitigation. To address this gap, we previously conducted a scoping review that identified 45 scientifically tested tools and applications aimed at mitigating bias,^16^ specifically focusing on racial and ethnic bias. Second, there is a dearth of knowledge regarding the effectiveness of these bias mitigation strategies. As outlined in its research protocol,^17^ the Agency for Healthcare Research and Quality published a systematic review that describes several strategies used in the literature^18^ while noting the difficulty of quantifying and extrapolating the results for broader use. Two other recent reviews conducted similar research—one review focused only on models using electronic health record data,^19^ and the other focused only on changes in racial/ethnic disparities.^20^

In the present review, we build on these efforts by identifying, categorizing, and describing the effectiveness of the bias reduction strategies and fairness metrics in healthcare algorithms, as articulated in our previous work. Specifically, we dissect studies from our scoping review that applied and evaluated bias mitigation algorithms and strategies to healthcare analytics in different care settings to examine qualitative and quantitative aspects of bias reduction approaches and metrics, the type of bias they address, and the stage at which these approaches are applied in the lifecycle of the healthcare algorithm. Our aim is to identify and ultimately recommend bias reduction approaches that are effective against algorithmic discrimination.

Our review includes a broader array of models trained on heterogeneous data than those examined in previous reviews^21–24^; as such, our review incorporates more bias types and places greater emphasis on advancing equity, an ethical principle that reflects our shared values as a society and provides the foundation on which other principles (ie, transparency, safety, regulatory compliance) can be effectively implemented. We also propose OPTIMIZE-AI^2^ (Optimizing Predictive Tools and Intelligence Models to Improve Zero Errors in AI [Accountability and Impact]), a framework to guide the development, evaluation, and deployment of equitable AI models in healthcare. To support their practical implementation, we relate these strategies to Duke Health’s Algorithm-Based Clinical Decision Support (ABCDS) Oversight,^25^ a framework that provides a structured approach for governing and evaluating clinical algorithms through 4 stages designed to guide development teams in the algorithmic lifecycle.

## Materials and methods

### Information sources

The databases searched include Medline (PubMed), Embase (Elsevier), and Web of Science Core Collection (Clarivate), ProQuest Computer Science Database, and ProQuest Dissertations & Theses Global.

### Definitions

We adopt definitions consistent with current guidance for AI in clinical prediction research. Artificial intelligence is defined following the TRIPOD+AI statement as a field of computer science that focuses on developing models and algorithms capable of performing tasks that typically require human intelligence.^26^ Algorithmic bias denotes systematic differences in the accuracy or calibration of predictive models across groups defined by protected characteristics such as race, sex, or age, and algorithmic fairness refers to clinical decision-making processes that do not consistently favor or disadvantage members of one protected class over another.^27^ To minimize ambiguity, we use “bias” to describe such systematic performance inequities rather than statistical estimation bias.

### Search strategy

This review categorizes and evaluates the effectiveness of bias reduction strategies and fairness metrics; the authors followed the same search strategy as that utilized in a previously conducted scoping review.^16^ The literature search was developed and conducted by an experienced medical librarian (LL) with input from the other authors and included a mix of keywords and subject headings: “algorithm,” “bias,” “mitigation/assessment,” “healthcare,” and “race/ethnicity.”

The original search, which was conducted on August 24, 2022, was extended but limited to studies published up to November 30, 2022, to capture the state of AI bias in healthcare prior to the widespread adoption of foundational models and transformer-based architectures, sparked by the public release of ChatGPT in 2022 (OpenAI, San Francisco, CA, United States), which introduced a new paradigm in AI development and application, known as generative AI.^28^^,^^29^ Empirically, analysis of Dimensions bibliometric data (Dimensions.ai, Digital Science, London, United Kingdom) shows that publications explicitly referencing “foundational model(s),” “large language model(s),” and related AI terminology increased more than 10-fold beginning in December 2022 (Figure S1).

The pre-foundational-model era reflects the state of bias mitigation prior to the proliferation of transformer-based architectures (eg, generative pretrained transformer and related models). Restricting our search to the pre-foundational-model era of healthcare AI enables a coherent synthesis of pre-generative AI mitigation strategies and establishes a foundation for subsequent reviews incorporating developments after 2022.

The full, reproducible search strategies for all included databases are provided in Supplementary Material S1.

### Eligibility criteria and study selection process

**Figure 1.**
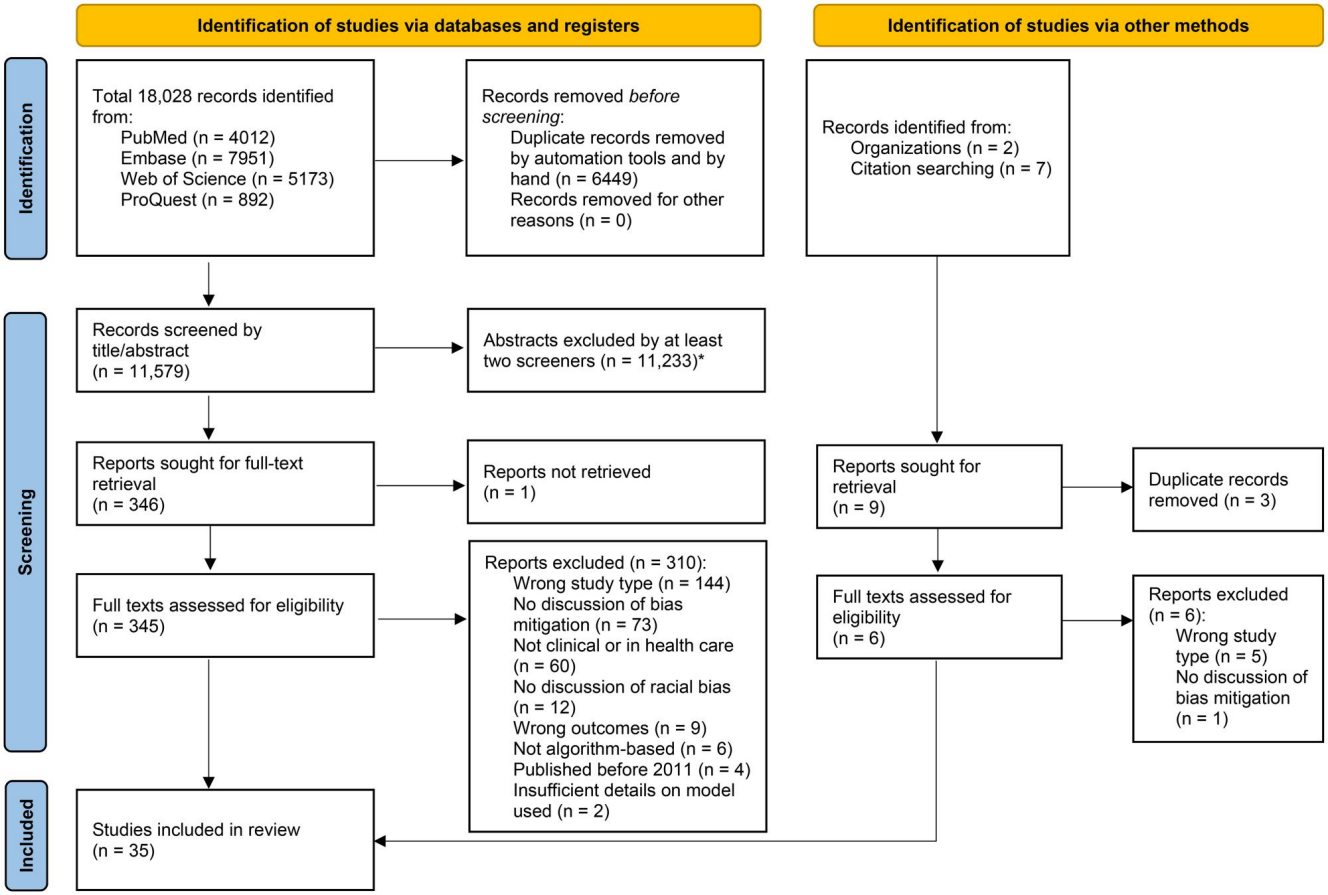
PRISMA diagram of the literature search and selection process. *Common reasons for exclusion at the title/abstract screening stage included (1) studies not related to bias mitigation in algorithmic models, (2) studies without clinical or population-level applications, (3) studies not in healthcare settings, and (4) studies lacking empirical data (eg, editorials, commentaries, conference abstracts).

We followed the Preferred Reporting Items for Systematic reviews and Meta-Analyses (PRISMA) guidelines and the same study selection process and inclusion criteria as those employed in the previous scoping review.^16^^,^^30^^,^^31^ To evaluate the effectiveness of the mitigation strategies reported in empirical studies, we added new exclusion criteria to the present systematic review: (1) studies not focusing on clinical applications and (2) studies with insufficient details on the model(s) used. Titles/abstracts and full texts were independently screened by at least 2 reviewers to exclude studies clearly outside the scope of this review, including those not addressing racial or ethnic bias mitigation, not involving algorithmic applications in healthcare, or representing non-empirical publications (eg, editorials, commentaries, or conference abstracts). Artificial intelligence tools were considered to be related to healthcare if they were algorithmic systems designed to inform or guide patient care or diagnostic, prognostic, or population health management decisions within clinical or public health settings. Any discrepancies between reviewers were resolved through discussion with a third reviewer. Detailed exclusion categories and counts for full-text screening are shown in Figure 1.

### Data collection and synthesis

Data extraction matrices were developed by M.P.C. and S.W. They included key components that helped define and contextualize the purpose, fairness metrics, and overall performance of an algorithm. All coauthors pilot-tested the extraction matrices on 2 studies. After team discussions, the matrices were revised, and an extraction manual was developed to ensure consistent and accurate coding for the full extraction phase. Two reviewers independently extracted data from each paper. Conflicts that were unresolvable between 2 reviewers were resolved by K.G Kais Gadhoumi.

### Quality/risk of bias assessment

We used the Standard Quality Assessment Criteria for Evaluating Primary Research Papers from a Variety of Fields (QualSys) tool to compare quality across the included studies (Table S1).^32^

### ABCDS framework

Duke Health’s ABCDS Oversight is a framework designed for the governance and evaluation of clinical algorithms.^25^ This framework ensures that these algorithms are innovative, safe, equitable, and high quality by introducing checkpoints throughout their development and deployment lifecycle. It involves 4 phases as follows. The Model Development phase necessarily includes clinical performance metrics for retrospective evaluation as well as the more traditional definition of model validation, for example, internal/external validation. The Silent Evaluation phase involves a prospective deployment of a model within a real-world clinical setting without any alerts generated for the clinical teams, which is used to evaluate model performance using real-life clinical data. The Effectiveness Evaluation phase involves small-scale deployment of the model for a subset of users, comparing its performance and user adoption to an existing standard. In the General Deployment phase, the model is deployed at a larger scale in typical clinical workflows with regular monitoring for performance drift or deviations.

## Results

### Summary of the included studies

Of the 18 028 studies identified from our scoping review,^16^ 11 579 unique records were screened, of which 346 studies were selected for full‐text retrieval and eligibility assessment. Nine additional studies were identified through targeted web searches and assessed against our eligibility criteria. After excluding 319 studies that failed to meet eligibility criteria, 35 studies were retained for inclusion. Figure 1 depicts the selection process and exclusion criteria flowchart following the PRISMA guidelines.

Table S2 provides a summary of the studies included. Of the 35 studies included in this review,^9^^,^^33–66^ all had an observational retrospective cohort design, and all but 6 (83%) were based on data from US sites; non-American sites included Israel, the United Kingdom, South Korea, and the Netherlands.^37^^,^^43^^,^^49^^,^^52^^,^^56^^,^^59^ Moreover, the included studies spanned various clinical settings across the care continuum, including inpatient, outpatient, labor and delivery, as well as academic and community acute care facilities. A few studies included in-person surveys^45^^,^^46^^,^^50^^,^^54^; they also encompassed many different clinical contexts and outcomes, such as inpatient mortality, pediatric postoperative mortality, cancer survival, pre-eclampsia, electrocardiogram analysis, and 30-day hospital readmission.

### Clinical categories

**Figure 2.**
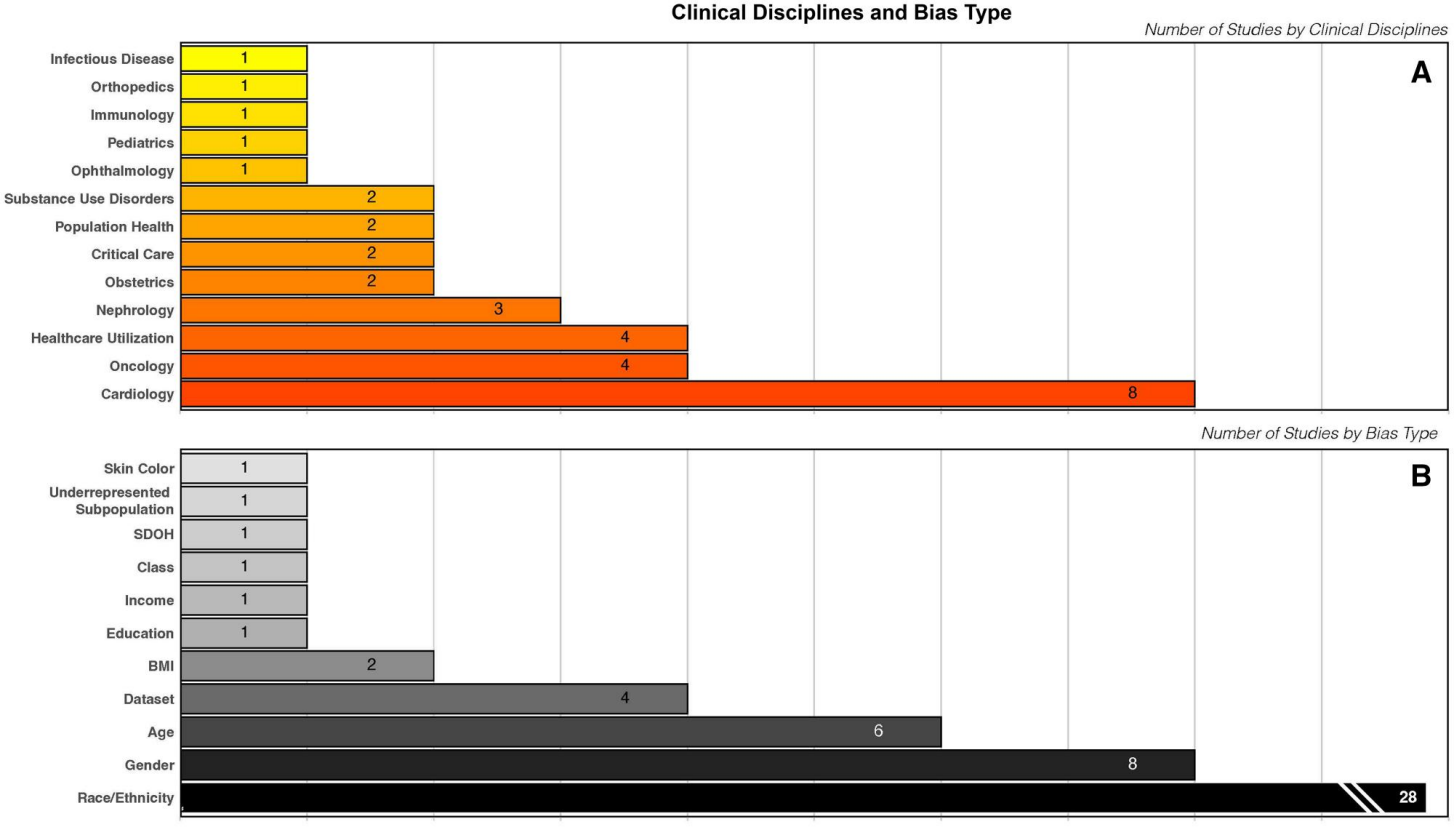
Distribution of studies by clinical subject area.

The studies evaluated bias and bias mitigation across multiple clinical subject areas, mostly in cardiology (23%), oncology (11%), healthcare utilization (11%), and nephrology (9%). Fewer studies involved other specialties and clinical settings, such as obstetrics (6%), critical care (6%), orthopedics (3%), and infectious disease (3%). Figure 2A depicts the number of studies by clinical subject area.

### Type of bias

The studies reported different types of bias. Racial and ethnic bias was the most frequently investigated form of bias in all studies (*n* = 28, 80%), followed by gender bias (*n* = 8, 23%) and age bias (*n* = 6, 17%). Most included studies used the terms “sex” and “gender” interchangeably in reference to bias without clearly defining or distinguishing between them. Other types of bias reported include colorism/shadeism, bias based on socioeconomic status, and bias toward underrepresented subpopulations. Figure 2B depicts the complete list of types of bias reported in the reviewed studies.

### Categorization of bias mitigation strategies

A variety of approaches for identifying and mitigating bias in clinical algorithms were reported (Table 1, Table S3). A useful categorization of approaches proposed in the literature is based on the stage of algorithm development at which they are applied. Pre-processing, in-processing, and post-processing approaches tackle debiasing at the algorithm data input, development, and output stages, respectively. In our previous scoping review,^16^ we proposed an additional category of “algorithmic design” specifically to implement the concept of *health equity by design*,^67^ embedding a fairness lens in the algorithm’s conceptualization phase rather than in its development phase.

**Table 1.** Bias mitigation approaches and techniques across the algorithmic lifecycle stages.

Type/stage	Approach	Technique	Studies
**Algorithm design**	Adjusted learning	Stratification of protected variables	Afrose et al.,^34^ Akbilgic et al.,^35^ Borgese et al.,^38^ Do et al.,^41^ Foryciarz et al.,^42^ Howard et al.,^47^ Noseworthy et al.,^53^ Puyol-Antón et al.,^59^ Segar et al.,^63^ and Segar et al.^62^
		Modification of outcome variables	Lin et al.,^51^ Obermeyer et al.,^9^ and Pierson et al.^58^
	Fairness through unawareness	Exclusion of protected variables	Buckley et al.,^39^ Gama et al.,^43^ Huang et al.,^48^ and Park et al.^55^
	Fairness through awareness	Inclusion of protected variables	Hammond et al.,^46^ Landy et al.,^50^ Pfohl et al.,^57^ Segar et al.,^62^ and Weissman et al.^66^
**Preprocessing**	Sampling/reweighing		Afrose et al.,^34^ Allen et al.,^36^ Burlina et al.,^40^ Mosteiro et al.,^52^ Park et al.,^55^ Pfohl et al.,^57^ Pierson et al.,^58^ Radovanović et al.,^61^ and Reeves et al.^60^
	Relabeling/perturbation		Park et al.^54^
**In-processing**	Regularization/constraints	Prejudice removal	Mosteiro et al.^52^ and Park et al.^55^
		Regularization through added bias-penalizing term	Perez Alday et al.^56^
	Adversarial learning		Adeli et al.^33^ and Radovanović et al.^61^
	Adjusted learning	Meta-learning	Puyol-Antón et al.^59^
		Modification of objective function	Do et al.^41^
		Transfer learning	Gao and Cui^44^ and Toseef et al.^65^
**Post-processing**	Output correction	Calibration	Barda et al.,^37^ Borgese et al.,^38^ Foryciarz et al.,^42^ Gianattasio et al.,^45^ Joo et al.,^49^ Pfohl et al.,^57^ and Thompson et al.^64^
	Model correction	Calibrated equalized odds	Radovanović et al.^61^

Because none of the included studies were evaluated in real-world clinical settings, all bias mitigation strategies and methods identified in this review focus on model development and fit within the Model Development phase of the ABCDS Oversight framework lifecycle. These strategies and methods are primarily implemented to enhance fairness and reduce bias during model creation and internal validation. Mitigation strategies applicable to subsequent phases of the ABCDS Oversight Framework, such as effectiveness evaluation, general deployment, and ongoing monitoring, were not observed in this review.

#### Algorithm design strategies

Twenty-one (60%) studies applied a bias mitigation strategy embedded in the design of the clinical algorithm. Several included the protected variables in the model,^46^^,^^50^^,^^57^^,^^62^^,^^66^ a concept known as *fairness through awareness*,^68^ while others excluded the protected variables,^39^^,^^43^^,^^48^^,^^55^ also known as *fairness through unawareness*.^69^ A few studies used an adjusted learning technique that modified the outcome variable^9^^,^^51^^,^^58^ or stratified the protected variables.^34^^,^^35^^,^^38^^,^^41^^,^^42^^,^^47^^,^^53^^,^^59^^,^^62^^,^^63^

#### Pre-processing strategies

Ten (29%) studies applied strategies prior to model training. Most studies applied a data resampling or reweighing technique to correct sampling bias by rebalancing the proportions of subgroups.^34^^,^^36^^,^^40^^,^^52^^,^^55^^,^^57^^,^^58^^,^^60^^,^^61^ One study used a disparate impact remover,^54^ a data perturbation technique that modifies attribute values to increase group fairness while preserving the rank order within groups.^70^

#### In-processing strategies

These strategies operate on the optimization procedure of the algorithm to achieve fairness and accuracy. Typically, this involves the modification of the objective function of the model through constraints and regularization, adversarial learning, or adjusted learning.^71–73^ Nine studies applied such strategies. Two studies^52^^,^^55^ applied a regularization approach based on prejudice removal,^74^ while one study added a bias-penalizing term to the loss function to achieve regularization and an ultimately fair outcome.^56^ Two other studies applied an adversarial learning approach, one by introducing a new adversarial loss function to minimize bias from protected variables^33^ and the other by using an adversarial neural network.^61^ Finally, 4 studies employed an adjusted learning approach to minimize bias: through meta-learning,^57^ through the modification of the objective function,^41^ or through the use of transfer learning.^44^^,^^65^

#### Post-processing strategies

Post-processing approaches act on and adjust the model outcome on a per-group basis, primarily by either correcting the outcome or by correcting the model itself. Seven studies (20%) corrected the outcome by calibration of the model output,^37^^,^^38^^,^^42^^,^^45^^,^^49^^,^^57^^,^^64^ and one study^61^ corrected the model by using a calibrated equalized odds technique.^75^

### Strategy evaluation: Fairness metrics

Numerous metrics were proposed to uncover bias, measure its effect, and quantify fairness improvement that debiasing strategies may accomplish.^76^ Fairness metrics serve the dual purpose of identifying and assessing bias in models, on the one hand, and evaluating the effectiveness of bias mitigation strategies, on the other. Fairness metrics extracted included demographic parity, equal opportunity, predictive parity, and calibration within groups. These metrics characterize equitable performance across demographic subgroups rather than overall model accuracy, emphasizing balanced utility for diverse patient populations. Although they differ on multiple levels, fairness metrics may be generally grouped into 2 main categories: group-based fairness metrics vs individual- and counterfactual-based fairness metrics^68^^,^^77^ (Supplementary Material S1). Notably, all 35 studies used group fairness metrics to evaluate the performance of the mitigation strategies they employed, and none of the studies employed an individual fairness metric.

Conceptually, group fairness metrics (Table 2) can be divided into 4 main categories that apply different but complementary statistical criteria to assess fairness in a decision support model^78^—parity-based, confusion matrix-based, calibration-based, and score-based metrics.^79^ Nine studies (26%) applied parity-based metrics^9^^,^^51^^,^^52^^,^^54^^,^^55^^,^^57^^,^^61^^,^^65^^,^^66^; 16 studies (46%) applied a version of an accuracy equality metric, which is a subtype of confusion matrix-based metric^38–41^^,^^43–45^^,^^47^^,^^48^^,^^50^^,^^53^^,^^54^^,^^56^^,^^58^^,^^62^^,^^64^; 8 studies (23%) used calibration as a fairness measure^9^^,^^37^^,^^38^^,^^42^^,^^48^^,^^49^^,^^57^^,^^63^; and only one study employed a score-based metric.^59^ A list of metrics reported by each study is provided in Table 2.

**Table 2.** Fairness metrics reported.

	Parity-based metrics	Confusion matrix-based metrics	Calibration-based metrics	Score-based metrics
**Definition**	Compare predicted positive rates across groups	Compare groups by considering potential underlying differences between groups	Compare groups based on predicted probability	Compare groups based on expected scores
**Statistical property tested**	Independence	Separation	Sufficiency	—
**Examples (studies)**	Statistical parity: Lin et al.,^51^ Mosteiro et al.,^52^ Obermeyer et al.,^9^ Park et al.,^54^ Pfohl et al.,^57^ Radovanović et al.^61^ Demographic parity: Toseef et al.,^65^ Weissman et al.,^66^ Disparate impact: Mosteiro et al.,^52^ Park et al.,^54^ Park et al.^55^	Accuracy equality: Borgese et al.,^38^ Buckley et al.,^39^ Burlina et al.,^40^ Do et al.,^41^ Gama et al.,^43^ Gao and Cui,^44^ Gianattasio et al.,^45^ Howard et al.,^47^ Huang et al.,^48^ Landy et al.,^50^ Noseworthy et al.,^53^ Park et al.,^54^ Perez Alday et al.,^56^ Pierson et al.,^58^ Segar et al.,^62^ Thompson et al.^64^ Equalized odds: Mosteiro et al.,^52^ Radovanović et al.,^61^ Reeves et al.^60^ Equal opportunity: Adeli et al.,^33^ Allen et al.,^36^ Hammond et al.,^46^ Lin et al.,^51^ Park et al.,^55^ Reeves et al.^60^	Test fairness, well calibration: Barda et al.,^37^ Borgese et al.,^38^ Foryciarz et al.,^42^ Huang et al.,^48^ Joo et al.,^49^ Obermeyer et al.,^9^ Pfohl et al.,^57^ Segar et al.^63^	Balance for positive and negative class, Bayesian fairness, statistical similarity: Puyol-Antón et al.^59^

No studies in the current review used individual- or counterfactual-based fairness metrics.

Parity-based metrics examine the statistical independence of protected variables and compare predicted positive rates across protected/sensitive groups. Examples of parity-based metrics include statistical parity, demographic parity, and disparate impact. Eleven of 35 (31%) studies applied parity-based metrics.

Confusion-based metrics measure statistical separation between protected variables and compare protected/sensitive groups by considering potential underlying differences between groups. Examples of such metrics include equalized odds, equalized opportunity, and accuracy equality. Many studies (16 of 35; 46%) applied a version of an accuracy equality metric (eg, by measuring the false discovery or omission rate instead of accuracy) to detect and mitigate bias.

Calibration-based metrics consider the statistical sufficiency of protected variables and compare outcome probability scores between groups to evaluate fairness. This definition of fairness may be met if participants with protected characteristics and those with unprotected characteristics are both equally likely to belong to the positive classification. Calibration, test fairness, and well calibration are examples of calibration-based metrics.^80^^,^^81^ Eight of the 35 studies (23%) used calibration to measure fairness.

Finally, score-based metrics compare protected groups based on expected scores. Examples of these metrics include statistical similarity, balance for positive and negative class, and Bayesian fairness. Only 1 of the 35 studies used a score-based metric.^59^

### Strategy evaluation: Effectiveness

**Figure 3.**
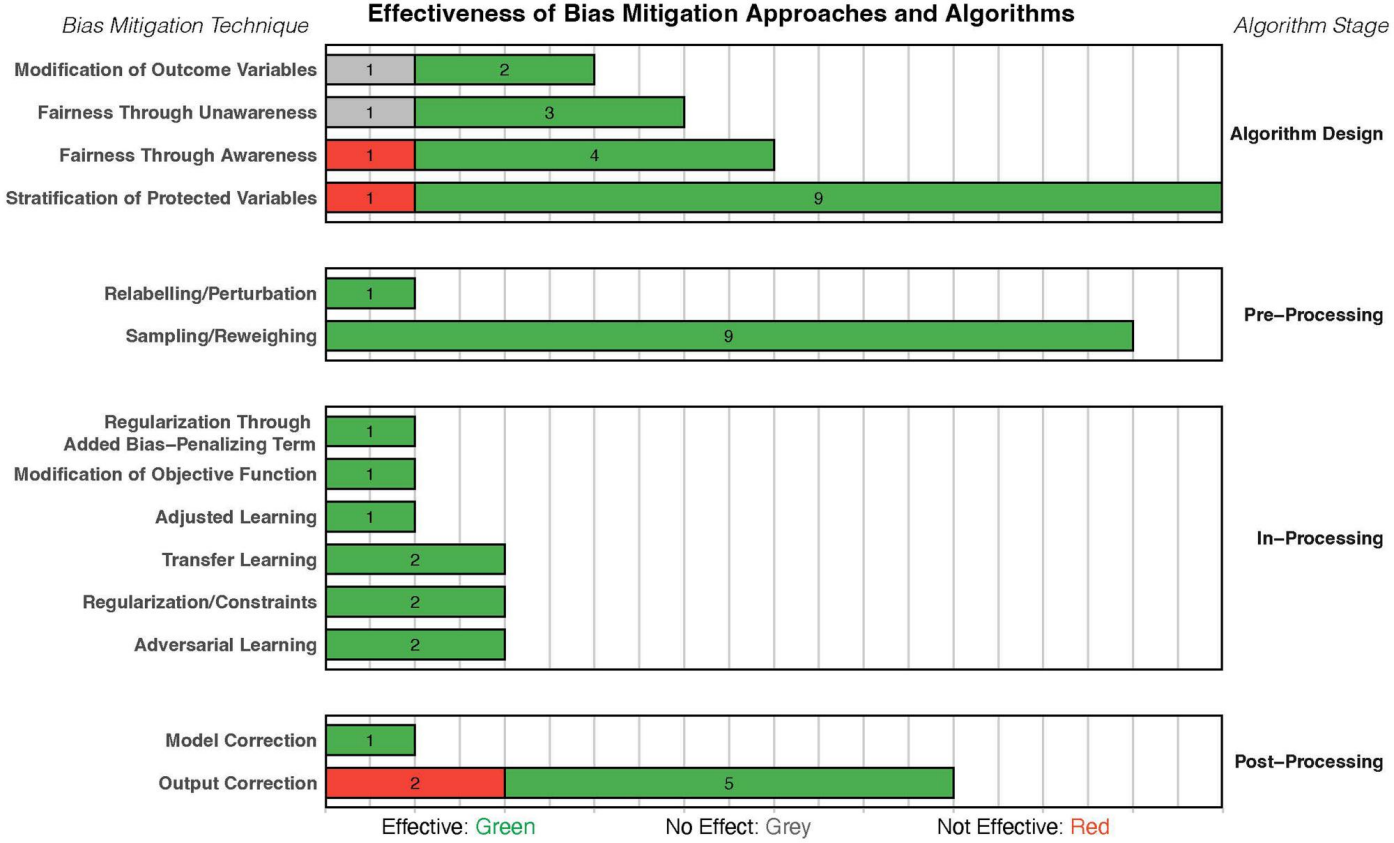
Bias mitigation strategies and their author-reported effectiveness.

Figure 3 summarizes the reported effectiveness of the bias mitigation strategies identified across the included studies. Importantly, in this context, “effectiveness” refers to author-reported effectiveness as described in the original publications. None of the included studies employed standardized or comparable definitions, criteria, or thresholds to evaluate bias reduction, and therefore, direct comparisons between methods or strategies are not possible. Likewise, no study reported stakeholder engagement or consultation to establish what would constitute a clinically meaningful reduction in algorithmic bias.

Most studies (86%) reported strategy effectiveness in terms of reducing bias or fairness improvement (eg, reduced subgroup disparity, improved model performance across subgroups). Only 3 studies (9%) reported ineffectiveness of strategies used,^38^^,^^64^^,^^66^ while 2 studies (6%) reported that no effect could be observed.^9^^,^^39^ Interestingly, the ineffective strategies used either algorithmic design approaches (eg, stratification of protected variables and inclusion of protected variables) or post-processing approaches (eg, calibration). All pre- and in-processing strategies were reported to be effective.

The specific outcomes and metrics used to report bias reduction in each study are detailed in Table S3.

## Discussion

In this study, we present a synthesis of 35 studies that explore various bias mitigation strategies and the application of fairness metrics designed to address bias in algorithms used for decision-making in healthcare. Our review includes a wide range of models trained on heterogeneous data to examine the contexts in which different strategies are utilized and their effectiveness, offering initial insight into potential relationships between the effectiveness of bias mitigation strategies and their clinical subject area. To support practical implementation, we aligned these strategies with the stages of Model Development, the first phase of the ABCDS Oversight framework lifecycle. Further, our review highlights the numerous decisions required to mitigate bias in clinical algorithms, such as selecting appropriate data pre-processing techniques, model optimization strategies, and post-processing adjustments tailored to specific contexts. The literature does not clearly define which clinical settings benefit most from each strategy, thereby highlighting the need for ongoing evaluation to better understand which approaches are most effective in different clinical scenarios.

### The intersection of bias reduction and fairness metrics

A fairness metric aims to evaluate the equity of model performance across different population subgroups rather than the overall clinical accuracy or quality of predictions. A model that demonstrates comparable predictive performance (eg, sensitivity, specificity, or predictive value) across groups defined by protected characteristics such as race, sex, or age may not necessarily be considered “fair.” All studies in this review assessed group fairness, typically by comparing model performance by demographic variables such as race, sex, or age. This predominant focus likely reflects the practical and regulatory alignment of group-level metrics, which are more established, interpretable, and feasible than individual or counterfactual fairness measures that require modeling unobservable patient-level scenarios. However, this emphasis may overlook inequities within groups and limit understanding of fairness at the individual level. Future work should extend fairness evaluation beyond group comparisons to incorporate individual and counterfactual perspectives for a more comprehensive and ethically grounded assessment of algorithmic equity in healthcare.

There is significant variability in the application of group-based fairness metrics, which highlights the complexity involved in selecting appropriate metrics for each context and the need for a deep understanding of both the metrics and the clinical environments they are applied in. Moreover, not all fairness metrics can be used with all bias mitigation strategies, as their compatibility depends on the type of model output, the stage at which mitigation is applied, and the specific fairness goals. Bias mitigation strategies, whether applied before, during, or after model training, are often designed to optimize specific fairness metrics, and improving one metric may compromise another. As a result, selecting appropriate combinations requires careful consideration of the clinical context and tradeoffs between fairness objectives.

No single bias mitigation approach has proven universally superior across all contexts.^82^ The effectiveness of a given strategy often depends on factors such as the dataset characteristics, model type, clinical application, and the specific definitions of fairness being prioritized. For example, methods that improve statistical parity may inadvertently reduce predictive accuracy or introduce new disparities across other subgroups.^75^^,^^83^^,^^84^ Similarly, fairness metrics can sometimes conflict with one another, making it challenging to optimize for all simultaneously.^57^^,^^85^ These tradeoffs underscore the importance of context-aware decision-making and highlight the need for ongoing evaluation, stakeholder engagement, and transparency when selecting and implementing bias mitigation approaches in healthcare AI.

### Bias reduction strategies lifecycle and effectiveness

Our categorization aligns model development processes (design, preprocessing, in-processing, and postprocessing) with the ABCDS lifecycle stages of Model Development. None of the included studies evaluated and mitigated bias in post-development phases (ie, Silent Evaluation, Effectiveness Evaluation, and General Deployment), as none had undergone prospective real-world testing. However, bias detection and mitigation should not end at the Model Development phase. Fairness assessment must be treated as an iterative and continuous process that extends throughout the entire lifecycle of algorithm-based clinical decision support systems. Current oversight and governance frameworks^25^^,^^86^^,^^87^ highlight the necessity of revisiting and updating bias assessments as models transition to prospective evaluation and postdeployment phases, where new or unanticipated biases may emerge due to shifts in data, clinical practice, or patient populations. Consistent with the ABCDS Oversight Framework, bias should be assessed at multiple entry points within each stage, and mitigation strategies should be adapted according to the type, source, and impact of the bias identified. This underscores the importance of viewing fairness as a dynamic, lifecycle-wide responsibility rather than a discrete activity confined to the Model Development phase. More broadly, the field must approach algorithmic tools with appropriate skepticism and demand rigorous, evidence-based prospective evaluation before integrating them into clinical practice. Postdeployment monitoring should only occur after robust predeployment validation to avoid repeating cycles of unrealistic expectations and unintended harm.^88^

Notably, while 85% of included studies reported positive or partially successful bias reduction, a small proportion (8% ineffective, 5% no effect) indicated limited or null results. This suggests potential publication bias favoring positive findings and underscores the importance of reporting neutral or negative outcomes to provide a full picture of mitigation efficacy. The few studies that achieved only partial bias reduction offer valuable insight into persistent challenges such as small sample sizes, limited subgroup representation, and lack of external validation. Recognizing these less successful efforts helps highlight methodological gaps in the current evidence base and underscores the need for more balanced reporting of both effective and ineffective bias mitigation approaches to strengthen transparency and reproducibility in future research. Moreover, assessing the magnitude of success in mitigating bias is notably challenging due to the absence of standardized methods and metrics. This diversity in methodologies and the lack of consensus on fairness metrics complicate the ability to compare the effectiveness of different strategies, thereby hindering a comprehensive understanding of their impact across various clinical contexts and algorithmic applications.

The interpretation of the “effectiveness” of mitigation strategies should be approached cautiously, as this term was based solely on author-reported results rather than standardized or externally validated criteria. The lack of uniform definitions and stakeholder involvement in evaluating what constitutes meaningful bias reduction limits the comparability and generalizability of these findings. Moving forward, developing consensus measures and reporting standards for assessing the effectiveness of bias-mitigation strategies will be critical to enable objective evaluation and translation of these approaches into clinical practice.

### Recommendations

Our findings emphasize that the strategic selection of strategies and fairness metrics is crucial to the success of bias mitigation efforts, thus directly influencing the assessment and enhancement of equity throughout different stages of an algorithm’s lifecycle. Practitioners should carefully consider the specific requirements and constraints of their application domain, as well as the potential tradeoffs between fairness and predictive performance. Importantly, bias-related concepts and terminologies should be employed with greater precision and transparency. Studies should adopt precise conceptual definitions and transparent methodological approaches to clearly differentiate biological sex from gender-related factors when assessing bias in healthcare algorithms.

To guide future development, deployment, and governance of AI models in healthcare, we propose the OPTIMIZE-AI^2^ framework. This framework provides a holistic approach to developing, deploying, and governing AI models in healthcare. It emphasizes outcome-focused evaluation, consistent performance across diverse groups, transparency, inclusivity, bias mitigation, seamless workflow integration, a zero-harm goal, continuous monitoring, and strong accountability structures. Together, these principles aim to ensure that AI systems are safe, equitable, effective, and aligned with ethical and regulatory standards. A detailed description of the OPTIMIZE-AI^2^ framework can be found in Supplementary Material S1.

### Limitations

This systematic review has several limitations that may affect the applicability and generalizability of its findings. First, there may be inherent patterns associated with certain features or variables that were not extracted, thus possibly causing the review to overlook nuanced data elements crucial for comprehensive bias analysis. Additionally, while our review mainly focused on the primary reported bias mitigation strategies, a few studies may have included secondary interventions that were not discussed in detail, thereby potentially underrepresenting the scope of efforts undertaken. A significant limitation arises from the measurement of fairness in many studies, which did not utilize validated or widely accepted fairness metrics. Instead, general metrics such as the area under the receiver operating characteristics curve, sensitivity, and specificity were often employed. While these metrics are useful for certain assessments, they do not provide a robust measure of fairness and may not accurately reflect biases in clinical algorithms.

Last, the studies included in this review were restricted to those published up to November 2022. This cutoff was intentionally selected to capture the landscape of bias in AI technologies in healthcare prior to a pivotal shift in the field. Empirical analysis of bibliometric records from Dimensions.ai (Digital Science, London, United Kingdom) indicates a pronounced and sustained surge in scholarly output referencing “foundational model(s),” “large language model(s),” and related generative AI terminology (Figure S1). Specifically, beginning in December 2022, the volume of publications containing these terms increased by more than an order of magnitude, reflecting a marked surge in research activity and dissemination within this domain. Around this time, the emergence and rapid adoption of foundational models, particularly large transformer-based architectures, marked a significant evolution in both AI capabilities and deployment strategies. These models introduced new methodological complexities, data dependencies, and ethical considerations that distinguish them from earlier AI systems. Including studies beyond this point would have introduced a qualitatively different set of technologies and challenges, necessitating a distinct analytic framework. Rather than mixing fundamentally distinct generations of AI technologies, this review focuses on the pre-foundational model era to provide a coherent and analytically consistent synthesis. Studying the pre-foundational-model period isolates traditional bias sources (ie, representation imbalance and model transparency) and provides a baseline for future comparative analyses. Although newer studies are not captured, this temporal boundary enhances the interpretability of our findings and lays the groundwork for future reviews that will address the implications of this new generation of AI technologies in healthcare.

## Conclusion

This systematic review highlighted the effectiveness of bias reduction strategies implemented during different stages of the algorithm lifecycle; the use of fairness metrics highlights their importance in promoting health equity and achieving the best possible health for all populations. For healthcare systems, these findings emphasize the need to critically evaluate the fairness of algorithms prior to their implementation and to continuously monitor their performance across diverse patient populations. Policymakers should consider developing guidelines for technology developers that mandate fairness assessments and bias mitigation strategies in the use of artificial intelligence in healthcare. Researchers are encouraged to include patients, families, and communities in codesign processes and employ multidisciplinary teams to develop fairness metrics and bias reduction techniques while continuously examining their impact on care delivery and outcomes. Developers, implementers, and regulators should interpret fairness evaluation as a continuous process integrated throughout the model lifecycle (from data collection to postdeployment surveillance) to maintain equitable and trustworthy AI in healthcare.

## Supplementary Material

ooag081_Supplementary_Data

## Data Availability

The data underlying this article are available in the article and in Supplementary Material S1.
