# Supplementary material for: Strategies for mitigating artificial intelligence bias in healthcare: a systematic review
Source: JAMIA Open. 2026 Jun 3;9(3):ooag081. doi: 10.1093/jamiaopen/ooag081 (PMC13234951; doi:10.1093/jamiaopen/ooag081)
Supplement: ooag081_Supplementary_Data [file ooag081_supplementary_data.docx]

**Supplementary Materials**

**Supplementary Glossary.** Glossary of Terms

**Supplementary Methods.** Literature Search Strategies

**Supplementary Discussion**. The OPTIMIZE-AI^2^ Framework

**Supplementary Table 1.** Quality Assessment Criteria for Quantitative Studies

**Supplementary Table 2.** Summary of the Included Studies

**Supplementary Table 3.**  Approaches to Identifying and Mitigating Bias in Clinical Algorithms

**Supplementary Figure 1.** Total Publications in Health, Clinical, and Biomedical Sciences Referencing Generative AI

**Supplementary Glossary. Glossary of Terms**

- **Algorithm-Based Clinical Decision Support (ABCDS) Framework**. The general framework used in the Duke University Health System for the development, deployment, and ongoing monitoring of machine learning algorithms in the clinical setting. The framework involves four phases as follows. The **Model Development** phase necessarily includes clinical performance metrics for retrospective evaluation as well as the more traditional definition of model validation, e.g. internal/external validation. The **Silent Evaluation** phase involves a prospective deployment of a model within a real-world clinical setting without any alerts generated for the clinical teams, which is used to evaluate model performance using real-life clinical data. The **Effectiveness Evaluation** phase involves small-scale deployment of the model for a subset of users, comparing its performance and user adoption to an existing standard. In the **General Deployment** phase, the model is deployed at a larger scale in typical clinical workflows with regular monitoring for performance drift or deviations.
- **Algorithm Stage**. As seen in Table 2, algorithm stage is defined in this work from the perspective of algorithm/model development. We specify whether a given bias mitigation strategy is implemented within the pre-processing, processing, or post-processing step (i.e. before, during, or after model training, respectively). We include “Algorithm Design” as a separate category within this framework to underscore the importance of early decisions in model development and their downstream effects on bias and fairness. Embracing the Office of the National Coordinator for Health Information Technology (ONC) “Health By Design” concept^1^, we emphasize the integral role that equity and fairness should have during model conceptualization and development.
- **Clinical Category**. The clinical specialty most commonly involved with the disease process or condition that the model is being used for. For example, a model predicting vaginal birth success after a prior Caesarean section would be listed under the “Obstetrics” category, while a model making predictions based on electrocardiograms would be under “Cardiology”.
- **Clinical Setting**. General level of care provision, e.g. inpatient, step-down, or skilled nursing facility; outpatient; etc.
- **Group-Based Fairness Metrics**. The category of fairness metrics that measure the parity of outcomes across protected/sensitive groups. They can be split into four main categories that apply different but complementary statistical criteria to assess fairness in a decision support model^2,3^:
  - **Calibration-based Fairness Metrics**. A sub-type of group-based fairness metric that considers the statistical sufficiency of protected variables and compares outcome probability scores between groups to evaluate fairness. Examples include calibration, test fairness, and well-calibration.^4,5^
  - **Confusion Matrix-based Fairness Metrics**. A sub-type of group-based fairness metric that measures statistical separation between protected variables and compares protected/sensitive groups by considering potential underlying differences between groups. Examples of such metrics include equalized odds, equalized opportunity, and accuracy equality.
  - **Parity-based Fairness Metrics**. A sub-type of group-based fairness metric that examines the statistical independence of protected variables and compares predicted positive rates across protected/sensitive groups. Examples include statistical parity, demographic parity, and disparate impact.
  - **Score-based Fairness Metrics**. A sub-type of group-based fairness metric that compares protected groups based on expected scores. Examples include statistical similarity, balance for positive and negative class, and Bayesian fairness.
- **Individual- or Counterfactual Fairness Metrics**. The category of fairness metrics that measure how well a model assigns similar outcomes to similar individuals, often using a similarity measure, irrespective of their group membership.
- **Protected Variables**. Sensitive variables of interest that relate to commonly protected demographic characteristics of an individual or population. Examples include age, gender, race/ethnicity, and income.
- **Rebalancing**: A type of bias mitigation strategy through which the model developer reweighs observations to guarantee fairness between different subgroups for a particular sensitive attribute. This strategy is typically employed during the pre-processing phase of model development.
- **Sample.** Description of the populations/samples used for model training purposes.

**References**

1. Argentieri R, Mason T, Hefcart J, Henry J. Embracing Health Equity by Design. Health IT Buzz. Published February 22, 2022. Accessed March 22, 2024. https://www.healthit.gov/buzz-blog/health-it/embracing-health-equity-by-design

2. Barocas S, Hardt M, Narayanan A. Fairness and Machine Learning: Limitations and Opportunities. MIT Press; 2023. https://books.google.com/books?id=HuGwEAAAQBAJ

3. Caton S, Haas C. Fairness in Machine Learning: A Survey. Published online 2020. doi:10.48550/ARXIV.2010.04053

4. Hardt M, Price E, Srebro N. Equality of Opportunity in Supervised Learning. Published online October 7, 2016. Accessed July 9, 2024. http://arxiv.org/abs/1610.02413

5. Kleinberg J, Mullainathan S, Raghavan M. Inherent Trade-Offs in the Fair Determination of Risk Scores. Published online November 17, 2016. Accessed July 9, 2024. http://arxiv.org/abs/1609.05807

**Supplementary Methods. Literature Search Strategies**

**Librarian Searcher:** Leila Ledbetter

**Peer-Reviewer:** Steph Hendren

**Date of completed search:** August 24, 2022

**Date of updated search:** N/A

**Total number of articles (before de-duplication): 18,028**

**Total number of articles (after de-duplication): 11,647**

**Database / Study Registry (including vendor/platform):** Medline (via PubMed)

| **Set #** | **Search Strategy** | **Results** |
| --- | --- | --- |
| 1  *Algorithms* | "Algorithms"[Mesh] OR "Artificial Intelligence"[Mesh:NoExp] OR "Machine Learning"[Mesh] OR "Deep Learning"[Mesh] OR Algorithm[tiab] OR Algorithms[tiab] OR Algorithmic[tiab] OR "Artificial Intelligence"[tiab] OR "Machine Learning"[tiab] OR "neural network"[tiab] OR "neural networks"[tiab] OR "deep learning"[tiab] OR "augmented intelligence"[tiab] OR "deep metric learning"[tiab] OR "Predictive model"[tiab] OR "predictive models"[tiab] OR "prediction model"[tiab] OR "prediction models"[tiab] OR AI[tiab] OR "risk score"[tiab] OR "risk scores"[tiab] OR "risk model"[tiab] OR "risk models"[tiab] | 752,687 |
| 2  Bias | "Bias"[Mesh] OR "Health Inequities"[Mesh] OR "Healthcare Disparities"[Mesh] OR "Health Status Disparities"[Mesh] OR "Race Factors"[Mesh] OR "Systemic Racism"[Mesh] OR "Racism"[Mesh] OR Biases[tiab] OR bias[tiab] OR biased[tiab] OR debias[tiab] OR debiased[tiab] OR "de-bias"[tiab] OR "de-biased"[tiab] OR racism[tiab] or racial[tiab] or racist[tiab] OR prejudice[tiab] OR prejudices[tiab] OR prejudiced[tiab] OR discriminate[tiab] OR discriminating[tiab] OR discriminates[tiab] OR discriminatory[tiab] OR discriminated[tiab] OR discrimination[tiab] OR stereotype[tiab] OR stereotypes[tiab] OR stereotyped[tiab] OR stereotyping[tiab] OR stereotypical[tiab] OR profiling[tiab] OR disparity[tiab] OR disparities[tiab] OR inequities[tiab] OR inequity[tiab] OR inequality[tiab] OR inequalities[tiab] OR equities[tiab] OR equity[tiab] OR equality[tiab] OR equalities[tiab] OR ethics[tiab] OR ethical[tiab] OR ethically[tiab] OR unethical[tiab] OR unethically [tiab] OR accountable[tiab] OR accountability[tiab] | 1,079,154 |
| 3  Healthcare | "Insurance, Health"[Mesh] OR "hospitals"[MeSH Terms] OR "Risk Assessment"[Mesh] OR "Risk Management"[Mesh] OR "Disease Management"[Mesh] OR "Medical Records Systems, Computerized"[Mesh] OR "Electronic Health Records"[Mesh] OR "Decision Support Systems, Clinical"[Mesh] OR "Delivery of Health Care"[Mesh] OR "Health Services"[Mesh] OR "Medicare"[Mesh] OR "Medicaid"[Mesh] OR Health[tiab] OR "health care"[tiab] OR healthcare[tiab] OR hospitals[tiab] OR hospital[tiab] OR "medical center"[tiab] OR "medical centers"[tiab] OR "Health systems"[tiab] OR "Health system"[tiab] OR "health-care"[tiab] OR medical[tiab] OR clinical[tiab] OR risk[tiab] OR "care management"[tiab] OR "Health Services"[tiab] OR "disease management"[tiab] OR "disease prevention"[tiab] OR "health insurance"[tiab] OR "Medicare"[tiab] OR Medicaid[tiab] OR uninsured[tiab] OR insured[tiab] OR ((electronic[tiab] OR computerized[tiab] OR automated[tiab] OR administrative[tiab]) AND (medical[tiab] or health[tiab]) AND (record[tiab] OR records[tiab])) | 10,437,508 |
| 4  Mitigation/  assessment/  tools/  frameworks | "Checklist"[Mesh] OR mitigate[tiab] OR mitigates[tiab] OR mitigated[tiab] OR mitigating[tiab] OR mitigation[tiab] OR reduce[tiab] OR reduction[tiab] OR reduced[tiab] OR reduces[tiab] OR reducing[tiab] OR diminish[tiab] OR diminished[tiab] OR diminishing[tiab] OR diminishes[tiab] OR diminishment[tiab] OR alleviate[tiab] OR alleviates[tiab] OR alleviated[tiab] OR alleviating[tiab] OR amend[tiab] OR correct[tiab] OR corrects[tiab] OR corrected[tiab] OR correcting[tiab] OR correction[tiab] OR rectify[tiab] OR rectifies[tiab] OR rectified[tiab] OR rectifying[tiab] OR rectification[tiab] OR reform[tiab] OR reforms[tiab] OR reformed[tiab] OR reforming[tiab] OR reformation[tiab] OR ameliorate[tiab] OR strategy[tiab] OR strategies[tiab] OR prevent[tiab] OR prevents[tiab] OR preventing[tiab] OR prevention[tiab] OR ensure[tiab] OR ensures[tiab] OR ensuring[tiab] OR ensured[tiab] OR detect[tiab] OR detects[tiab] OR detected[tiab] OR detecting[tiab] OR detection[tiab] OR limit[tiab] OR limits[tiab] OR limited[tiab] OR limiting[tiab] OR limitation[tiab] OR limitations[tiab] OR avoid[tiab] OR avoids[tiab] OR avoided[tiab] OR avoiding[tiab] OR remove[tiab] OR removes[tiab] OR removed[tiab] OR removing[tiab] OR removal[tiab] OR decrease[tiab] OR decreases[tiab] OR decreased[tiab] OR decreasing[tiab] OR address[tiab] OR addresses[tiab] OR addressed[tiab] OR addressing[tiab] OR combat[tiab] OR combats[tiab] OR combated[tiab] OR combatted[tiab] OR combatting[tiab] OR eliminate[tiab] OR eliminates[tiab] OR eliminated[tiab] OR eliminating[tiab] OR elimination[tiab] OR minimize[tiab] OR minimizes[tiab] OR minimized[tiab] OR minimizing[tiab] OR minimise[tiab] OR minimises[tiab] OR minimised[tiab] OR minimising[tiab] OR Assess[tiab] OR assesses[tiab] OR assessed[tiab] OR assessing[tiab] OR assessment[tiab] OR evaluate[tiab] OR evaluates[tiab] OR evaluated[tiab] OR evaluating[tiab] OR evaluation[tiab] OR framework[tiab] OR frameworks[tiab] OR validate[tiab] OR validates[tiab] OR validated[tiab] OR validating[tiab] OR validation[tiab] OR measure[tiab] OR measures[tiab] OR measured[tiab] OR measuring[tiab] OR measurement[tiab] OR monitor[tiab] OR monitors[tiab] OR monitored[tiab] OR monitoring[tiab] OR analyze[tiab] OR analyzes[tiab] OR analyzed[tiab] OR analyzing[tiab] OR analysis[tiab] OR analyse[tiab] OR analyses[tiab] OR analysed[tiab] OR analysing[tiab] OR screen[tiab] OR screens[tiab] OR screened[tiab] OR screening[tiab] OR report[tiab] OR reports[tiab] OR reported[tiab] OR reporting[tiab] OR compare[tiab] OR compares[tiab] OR compared[tiab] OR comparing[tiab] OR comparison[tiab] OR diagnose[tiab] OR diagnoses[tiab] OR diagnosed[tiab] OR diagnosing[tiab] OR diagnostic[tiab] OR diagnosis[tiab] OR audit[tiab] OR audits[tiab] OR auditing[tiab] OR audited[tiab] OR comply[tiab] OR complies[tiab] OR complied[tiab] OR complying[tiab] OR compliance[tiab] OR validate[tiab] OR validates[tiab] OR validated[tiab] OR validating[tiab] OR validation[tiab] OR "algorithmic hygiene"[tiab] OR "algorithmic playbook"[tiab] OR "algorithmic bias playbook"[tiab] OR checklist[tiab] OR checklists[tiab] OR tool[tiab] OR tools[tiab] OR toolkit[tiab] OR toolkits[tiab] OR guideline[tiab] OR guidelines[tiab] OR rubric[tiab] OR rubrics[tiab] OR guidance[tiab] OR standard[tiab] OR standards[tiab] OR method[tiab] OR methods[tiab] OR methodology[tiab] OR methodologies[tiab] OR checkpoint[tiab] OR checkpoints[tiab] OR recommendation[tiab] OR recommendations[tiab] OR benchmark[tiab] OR benchmarks[tiab] OR regulation[tiab] OR regulations[tiab] OR regulatory[tiab] OR generalizable[tiab] OR generalizability[tiab] OR "prejudice remover"[tiab] OR fair[tiab] OR unfair[tiab] OR fairness[tiab] OR unfairness[tiab] OR "classification parity"[tiab] OR trust[tiab] OR trustworthy[tiab] OR trustworthiness[tiab] OR PROBAST[tiab] OR "Prediction Model Risk Of Bias Assessment Tool"[tiab] OR "Minimum Information for Medical AI Reporting guideline"[tiab] OR "Artificial Intelligence Risk Management Framework"[tiab] OR "AI RMF"[tiab] OR "Medical Information Mart for Intensive Care"[tiab] OR MIMIC[tiab] OR "balanced accuracy"[tiab] OR "disparate impact"[tiab] OR "equal opportunity"[tiab] OR "equalized odds"[tiab] OR "statistical parity"[tiab] | 22,393,661 |
| 5  Race/ethnicity | "Ethnicity"[Mesh] OR "Minority Health"[Mesh] OR "Ethnic and Racial Minorities"[Mesh] OR "Blacks"[Mesh] OR "African Americans"[Mesh] OR "Mexican Americans"[Mesh] OR "Hispanic or Latino"[Mesh] OR "Indians, North American"[Mesh] OR "American Indians or Alaska Natives"[Mesh] OR "Indigenous Peoples"[Mesh] OR "Asian Americans"[Mesh] OR "Native Hawaiian or Other Pacific Islander"[Mesh] OR "Transients and Migrants"[Mesh] OR "Emigrants and Immigrants"[Mesh] OR "minority group"[tiab] OR ethnic[tiab] OR ethnicity[tiab] OR "minority groups"[tiab] OR "minority population"[tiab] OR "minority populations"[tiab] OR "people of color"[tiab] OR "person of color"[tiab] OR BIPOC[tiab] OR "minority health"[tiab] OR race[tiab] OR races[tiab] OR racial[tiab] OR racially[tiab] OR "African Americans"[tiab] OR "African ancestry"[tiab] OR Black[tiab] OR Blacks[tiab] OR "African American"[tiab] OR "Hispanic American"[tiab] OR "Hispanic-American"[tiab] OR "Hispanic Americans"[tiab] OR "Hispanic-Americans"[tiab] OR Hispanics[tiab] OR Hispanic[tiab] OR "Mexican American"[tiab] OR "Mexican-American"[tiab] OR "Mexican Americans"[tiab] OR "Mexican-Americans"[tiab] OR "Cuban American"[tiab] OR "Cuban-American"[tiab] OR "Cuban Americans"[tiab] OR "Cuban-Americans"[tiab] OR "Latin American"[tiab] OR "Latin-American"[tiab] OR "Latin Americans"[tiab] OR "Latin-Americans"[tiab] OR Latinos[tiab] OR Latino[tiab] OR Latinas[tiab] OR Latina[tiab] OR Latinx[tiab] OR latine[tiab] OR latines[tiab] OR "Spanish speaking"[tiab] OR "Spanish-speaking"[tiab] OR "Spanish speakers"[tiab] OR "Spanish-speakers"[tiab] OR Mexican[tiab] OR Mexicans[tiab] OR "Puerto Rican"[tiab] OR "Puerto Ricans"[tiab] OR "Asian American"[tiab] OR "Asian Americans"[tiab] OR Asian[tiab] OR "Japanese Americans"[tiab] OR "Japanese American"[tiab] OR "Chinese Americans"[tiab] OR "Chinese American"[tiab] OR "Vietnamese Americans"[tiab] OR "Vietnamese American"[tiab] OR "Asian Indian Americans"[tiab] OR "Asian Indian American"[tiab] OR "Cambodian Americans"[tiab] OR "Cambodian American"[tiab] OR "Hmong Americans"[tiab] OR "Hmong American"[tiab] OR "Korean Americans"[tiab] OR "Korean American"[tiab] OR "Filipino Americans"[tiab] OR "Filipino American"[tiab] OR "Indigenous People"[tiab] OR "Indigenous People"[tiab] OR "Indigenous Population"[tiab] OR "Indigenous Populations"[tiab] OR tribes[tiab] OR "Native Peoples"[tiab] OR "Native People"[tiab] OR "First Nation"[tiab] OR "native person"[tiab] OR Alaskan[tiab] OR Hawaiian[tiab] OR Alaskans[tiab] OR Hawaiians[tiab] OR "Caribbean American"[tiab] OR "Caribbean-American"[tiab] OR "Caribbean Americans"[tiab] OR "Caribbean-Americans"[tiab] OR migrant[tiab] OR migrants[tiab] OR immigrant[tiab] OR immigrants[tiab] OR emigrant[tiab] OR emigrants[tiab] OR demographic[tiab] OR demographics[tiab] OR Polynesians[tiab] OR Polynesian[tiab] OR "pacific islander"[tiab] OR "pacific islanders"[tiab] | 1,056,938 |
| 6 | #1 AND #2 AND #3 AND #4 AND #5 | 4,024 |
| 7  Remove animal studies | #6 NOT (animals[MeSH Terms] NOT humans[MeSH Terms]) | 4,012 |

2^nd^ Database: Embase (via Elsevier)

| **Set #** | **Search Strategy** | **Results** |
| --- | --- | --- |
| 1  Algorithms | 'Algorithm'/de OR 'Algorithm bias'/de OR 'Artificial Intelligence'/de OR 'automated reasoning'/de OR 'Machine Learning'/exp OR 'Deep Learning'/de OR 'predictive model'/de OR 'risk model'/de OR Algorithm:ti,ab OR Algorithms:ti,ab OR Algorithmic:ti,ab OR 'Artificial Intelligence':ti,ab OR 'Machine Learning':ti,ab OR 'deep learning':ti,ab OR 'augmented intelligence':ti,ab OR 'deep metric learning':ti,ab OR 'Predictive model':ti,ab OR 'predictive models':ti,ab OR 'prediction model':ti,ab OR 'prediction models':ti,ab OR AI:ti,ab OR 'risk score':ti,ab OR 'risk scores':ti,ab OR 'risk model':ti,ab OR 'risk models':ti,ab | 921,128 |
| 2  Bias | 'Prejudice'/de OR 'Health Disparity'/de OR 'Race'/de OR 'Racism'/exp OR 'Implicit Bias'/de OR Biases:ti,ab OR bias:ti,ab OR biased:ti,ab OR debias:ti,ab OR debiased:ti,ab OR 'de-bias':ti,ab OR 'de-biased':ti,ab OR racism:ti,ab OR racial:ti,ab OR racist:ti,ab OR prejudice:ti,ab OR prejudices:ti,ab OR prejudiced:ti,ab OR discriminate:ti,ab OR discriminating:ti,ab OR discriminates:ti,ab OR discriminatory:ti,ab OR discriminated:ti,ab OR discrimination:ti,ab OR stereotype:ti,ab OR stereotypes:ti,ab OR stereotyped:ti,ab OR stereotyping:ti,ab OR stereotypical:ti,ab OR profiling:ti,ab OR disparity:ti,ab OR disparities:ti,ab OR inequities:ti,ab OR inequity:ti,ab OR inequality:ti,ab OR inequalities:ti,ab OR equities:ti,ab OR equity:ti,ab OR equality:ti,ab OR equalities:ti,ab OR ethics:ti,ab OR ethical:ti,ab OR ethically:ti,ab OR unethical:ti,ab OR unethically:ti,ab OR accountable:ti,ab OR accountability:ti,ab | 1,349,533 |
| 3  Healthcare | 'Health Insurance'/de OR hospital/exp OR 'Risk Assessment'/exp OR 'Risk Management'/de OR 'Disease Management'/de OR 'Electronic Medical Records System'/de OR 'Health Care delivery'/de OR 'Health Services'/de OR 'Health care'/de OR Medicare/de OR Medicaid/de OR 'electronic health record'/exp OR Health:ti,ab OR 'health care':ti,ab OR healthcare:ti,ab OR hospitals:ti,ab OR hospital:ti,ab OR 'medical center':ti,ab OR 'medical centers':ti,ab OR 'Health systems':ti,ab OR 'Health system':ti,ab OR health-care:ti,ab OR medical:ti,ab OR clinical:ti,ab OR risk:ti,ab OR 'care management':ti,ab OR 'Health Services':ti,ab OR 'disease management':ti,ab OR 'disease prevention':ti,ab OR 'health insurance':ti,ab OR Medicare:ti,ab OR Medicaid:ti,ab OR uninsured:ti,ab OR insured:ti,ab OR 'electronic health records':ti,ab OR ((electronic OR computerized OR automated OR administrative) NEAR/2 (medical OR health) NEAR/2 (records OR records)):ti,ab | 13,131,397 |
| 4  Mitigation/  assessment | 'checklist'/exp OR mitigate:ti,ab OR mitigates:ti,ab OR mitigated:ti,ab OR mitigating:ti,ab OR mitigation:ti,ab OR reduce:ti,ab OR reduction:ti,ab OR reduced:ti,ab OR reduces:ti,ab OR reducing:ti,ab OR diminish:ti,ab OR diminished:ti,ab OR diminishing:ti,ab OR diminishes:ti,ab OR diminishment:ti,ab OR alleviate:ti,ab OR alleviates:ti,ab OR alleviated:ti,ab OR alleviating:ti,ab OR amend:ti,ab OR amends:ti,ab OR amended:ti,ab OR amending:ti,ab OR correct:ti,ab OR corrects:ti,ab OR corrected:ti,ab OR correcting:ti,ab OR correction:ti,ab OR rectify:ti,ab OR rectifies:ti,ab OR rectified:ti,ab OR rectifying:ti,ab OR rectification:ti,ab OR reform:ti,ab OR reforms:ti,ab OR reformed:ti,ab OR reforming:ti,ab OR reformation:ti,ab OR ameliorate:ti,ab OR strategy:ti,ab OR strategies:ti,ab OR prevent:ti,ab OR prevents:ti,ab OR preventing:ti,ab OR prevention:ti,ab OR ensure:ti,ab OR ensures:ti,ab OR ensuring:ti,ab OR ensured:ti,ab OR detect:ti,ab OR detects:ti,ab OR detected:ti,ab OR detecting:ti,ab OR detection:ti,ab OR limit:ti,ab OR limits:ti,ab OR limited:ti,ab OR limiting:ti,ab OR limitation:ti,ab OR limitations:ti,ab OR avoid:ti,ab OR avoids:ti,ab OR avoided:ti,ab OR avoiding:ti,ab OR remove:ti,ab OR removes:ti,ab OR removed:ti,ab OR removing:ti,ab OR removal:ti,ab OR decrease:ti,ab OR decreases:ti,ab OR decreased:ti,ab OR decreasing:ti,ab OR address:ti,ab OR addresses:ti,ab OR addressed:ti,ab OR addressing:ti,ab OR combat:ti,ab OR combats:ti,ab OR combated:ti,ab OR combatted:ti,ab OR combatting:ti,ab OR eliminate:ti,ab OR eliminates:ti,ab OR eliminated:ti,ab OR eliminating:ti,ab OR elimination:ti,ab OR minimize:ti,ab OR minimizes:ti,ab OR minimized:ti,ab OR minimizing:ti,ab OR minimise:ti,ab OR minimises:ti,ab OR minimised:ti,ab OR minimising:ti,ab OR Assess:ti,ab OR assesses:ti,ab OR assessed:ti,ab OR assessing:ti,ab OR assessment:ti,ab OR evaluate:ti,ab OR evaluates:ti,ab OR evaluated:ti,ab OR evaluating:ti,ab OR evaluation:ti,ab OR framework:ti,ab OR frameworks:ti,ab OR validate:ti,ab OR validates:ti,ab OR validated:ti,ab OR validating:ti,ab OR validation:ti,ab OR measure:ti,ab OR measures:ti,ab OR measured:ti,ab OR measuring:ti,ab OR measurement:ti,ab OR monitor:ti,ab OR monitors:ti,ab OR monitored:ti,ab OR monitoring:ti,ab OR analyze:ti,ab OR analyzes:ti,ab OR analyzed:ti,ab OR analyzing:ti,ab OR analysis:ti,ab OR analyse:ti,ab OR analyses:ti,ab OR analysed:ti,ab OR analysing:ti,ab OR screen:ti,ab OR screens:ti,ab OR screened:ti,ab OR screening:ti,ab OR report:ti,ab OR reports:ti,ab OR reported:ti,ab OR reporting:ti,ab OR compare:ti,ab OR compares:ti,ab OR compared:ti,ab OR comparing:ti,ab OR comparison:ti,ab OR diagnose:ti,ab OR diagnoses:ti,ab OR diagnosed:ti,ab OR diagnosing:ti,ab OR diagnostic:ti,ab OR diagnosis:ti,ab OR audit:ti,ab OR audits:ti,ab OR auditing:ti,ab OR audited:ti,ab OR comply:ti,ab OR complies:ti,ab OR complied:ti,ab OR complying:ti,ab OR compliance:ti,ab OR validate:ti,ab OR validates:ti,ab OR validated:ti,ab OR validating:ti,ab OR validation:ti,ab OR 'algorithmic hygiene':ti,ab OR 'algorithmic playbook':ti,ab OR 'algorithmic bias playbook':ti,ab OR checklist:ti,ab OR checklists:ti,ab OR tool:ti,ab OR tools:ti,ab OR toolkit:ti,ab OR toolkits:ti,ab OR guideline:ti,ab OR guidelines:ti,ab OR rubric:ti,ab OR rubrics:ti,ab OR guidance:ti,ab OR standard:ti,ab OR standards:ti,ab OR method:ti,ab OR methods:ti,ab OR methodology:ti,ab OR methodologies:ti,ab OR checkpoint:ti,ab OR checkpoints:ti,ab OR recommendation:ti,ab OR recommendations:ti,ab OR benchmark:ti,ab OR benchmarks:ti,ab OR regulation:ti,ab OR regulations:ti,ab OR regulatory:ti,ab OR generalizable:ti,ab OR generalizability:ti,ab OR 'prejudice remover':ti,ab OR fair:ti,ab OR unfair:ti,ab OR fairness:ti,ab OR unfairness:ti,ab OR 'classification parity':ti,ab OR trust:ti,ab OR trustworthy:ti,ab OR trustworthiness:ti,ab OR PROBAST:ti,ab OR 'Prediction Model Risk Of Bias Assessment Tool':ti,ab OR 'Minimum Information for Medical AI Reporting guideline':ti,ab OR 'Artificial Intelligence Risk Management Framework':ti,ab OR 'AI RMF':ti,ab OR 'Medical Information Mart for Intensive Care':ti,ab OR MIMIC:ti,ab OR 'balanced accuracy':ti,ab OR 'disparate impact':ti,ab OR 'equal opportunity':ti,ab OR 'equalized odds':ti,ab OR 'statistical parity':ti,ab | 28,945,812 |
| 5  Race/  ethnicity | Ethnicity/de OR 'Minority Health'/de OR 'Ethnic group'/de OR 'minority group'/de OR 'Black person'/exp OR 'African Americans'/de OR 'Mexican Americans'/de OR 'Hispanic'/exp OR 'Puerto Rican'/de OR 'Asian Americans'/de OR 'American Indian'/de OR 'Alaska Native'/de OR 'Indigenous People'/de OR 'First Nation'/de OR 'Migrant'/exp OR 'demographics'/exp OR 'Polynesian'/exp OR 'Pacific Islander'/exp OR 'minority group':ti,ab OR ethnic:ti,ab OR ethnicity:ti,ab OR 'minority groups':ti,ab OR 'minority population':ti,ab OR 'minority populations':ti,ab OR 'people of color':ti,ab OR 'person of color':ti,ab OR BIPOC:ti,ab OR 'minority health':ti,ab OR race:ti,ab OR races:ti,ab OR racial:ti,ab OR racially:ti,ab OR 'African Americans':ti,ab OR 'African ancestry':ti,ab OR Black:ti,ab OR Blacks:ti,ab OR 'African American':ti,ab OR 'Hispanic American':ti,ab OR Hispanic-American:ti,ab OR 'Hispanic Americans':ti,ab OR Hispanic-Americans:ti,ab OR Hispanics:ti,ab OR Hispanic:ti,ab OR 'Mexican American':ti,ab OR Mexican-American:ti,ab OR 'Mexican Americans':ti,ab OR Mexican-Americans:ti,ab OR 'Cuban American':ti,ab OR Cuban-American:ti,ab OR 'Cuban Americans':ti,ab OR Cuban-Americans:ti,ab OR 'Latin American':ti,ab OR Latin-American:ti,ab OR 'Latin Americans':ti,ab OR Latin-Americans:ti,ab OR Latinos:ti,ab OR Latino:ti,ab OR Latinas:ti,ab OR Latina:ti,ab OR Latinx:ti,ab OR latine:ti,ab OR latines:ti,ab OR 'Spanish speaking':ti,ab OR Spanish-speaking:ti,ab OR 'Spanish speakers':ti,ab OR Spanish-speakers:ti,ab OR Mexican:ti,ab OR Mexicans:ti,ab OR 'Puerto Rican':ti,ab OR 'Puerto Ricans':ti,ab OR 'Asian American':ti,ab OR 'Asian Americans':ti,ab OR Asian:ti,ab OR 'Japanese Americans':ti,ab OR 'Japanese American':ti,ab OR 'Chinese Americans':ti,ab OR 'Chinese American':ti,ab OR 'Vietnamese Americans':ti,ab OR 'Vietnamese American':ti,ab OR 'Asian Indian Americans':ti,ab OR 'Asian Indian American':ti,ab OR 'Cambodian Americans':ti,ab OR 'Cambodian American':ti,ab OR 'Hmong Americans':ti,ab OR 'Hmong American':ti,ab OR 'Korean Americans':ti,ab OR 'Korean American':ti,ab OR 'Filipino Americans':ti,ab OR 'Filipino American':ti,ab OR 'Indigenous People':ti,ab OR 'Indigenous People':ti,ab OR 'Indigenous Population':ti,ab OR 'Indigenous Populations':ti,ab OR tribes:ti,ab OR 'Native Peoples':ti,ab OR 'Native People':ti,ab OR 'First Nation':ti,ab OR 'native person':ti,ab OR Alaskan:ti,ab OR Alaskans:ti,ab OR 'Caribbean American':ti,ab OR Caribbean-American:ti,ab OR 'Caribbean Americans':ti,ab OR Caribbean-Americans:ti,ab OR migrant:ti,ab OR migrants:ti,ab OR immigrant:ti,ab OR immigrants:ti,ab OR emigrant:ti,ab OR emigrants:ti,ab OR demographic:ti,ab OR demographics:ti,ab OR Polynesians:ti,ab OR Polynesian:ti,ab OR 'pacific islander':ti,ab OR 'pacific islanders':ti,ab OR Hawaiian:ti,ab OR Hawaiians:ti,ab | 1,548,451 |
| 6 | #1 AND #2 AND #3 AND #4 AND #5 | 8,230 |
| 7 | #6 AND [humans]/lim | 7,951 |

3rd Database: Web of Science (Clarivate)

| **Set #** | **Search Strategy** | **Results** |
| --- | --- | --- |
| 1  Algorithms | TS=("Artificial Intelligence" OR "Machine Learning" OR "Deep Learning" OR Algorithm OR Algorithms OR Algorithmic OR "augmented intelligence" OR "deep metric learning" OR "Predictive model" OR "predictive models" OR "prediction model" OR "prediction models" OR AI OR "risk score" OR "risk scores" OR "risk model" OR "risk models") | 2,913,410 |
| 2  Bias | TS=("Health Status Disparities" OR "Race Factors" OR Biases OR bias OR biased OR debias OR debiased OR "de-bias" OR "de-biased" OR racism OR racial OR racist OR prejudice OR prejudices OR prejudiced OR discriminate OR discriminating OR discriminates OR discriminatory OR discriminated OR discrimination OR stereotype OR stereotypes OR stereotyped OR stereotyping OR stereotypical OR profiling OR disparity OR disparities OR inequities OR inequity OR inequality OR inequalities OR equities OR equity OR equality OR equalities OR ethics OR ethical OR ethically OR unethical OR unethically OR accountable OR accountability) | 3,804,114 |
| 3  Healthcare | TS=("Health Insurance" OR "Risk Assessment" OR "Risk Management" OR "Disease Management" OR "Medical Records Systems" OR "Delivery of Health Care" OR "Health Services" OR Health OR "health care" OR healthcare OR hospitals OR hospital OR "medical center" OR "medical centers" OR "Health systems" OR "Health system" OR health-care OR medical OR risk OR clinical OR "care management" OR "disease prevention" OR Medicare OR Medicaid OR uninsured OR insured OR "Electronic Health Records" OR ((electronic OR computerized OR automated OR administrative) AND (medical or health) AND (record OR records))) | 10,034,108 |
| 4  Mitigation/  assessment | TS=(mitigate OR mitigates OR mitigated OR mitigating OR mitigation OR reduce OR reduction OR reduced OR reduces OR reducing OR diminish OR diminished OR diminishing OR diminishes OR diminishment OR alleviate OR alleviates OR alleviated OR alleviating OR amend OR amends OR amended OR amending OR correct OR corrects OR corrected OR correcting OR correction OR rectify OR rectifies OR rectified OR rectifying OR rectification OR reform OR reforms OR reformed OR reforming OR reformation OR ameliorate OR strategy OR strategies OR prevent OR prevents OR preventing OR prevention OR ensure OR ensures OR ensuring OR ensured OR detect OR detects OR detected OR detecting OR detection OR limit OR limits OR limited OR limiting OR limitation OR limitations OR avoid OR avoids OR avoided OR avoiding OR remove OR removes OR removed OR removing OR removal OR decrease OR decreases OR decreased OR decreasing OR address OR addresses OR addressed OR addressing OR combat OR combats OR combated OR combatted OR combatting OR eliminate OR eliminates OR eliminated OR eliminating OR elimination OR minimize OR minimizes OR minimized OR minimizing OR minimise OR minimises OR minimised OR minimising OR Assess OR assesses OR assessed OR assessing OR assessment OR evaluate OR evaluates OR evaluated OR evaluating OR evaluation OR framework OR frameworks OR validate OR validates OR validated OR validating OR validation OR measure OR measures OR measured OR measuring OR measurement OR monitor OR monitors OR monitored OR monitoring OR analyze OR analyzes OR analyzed OR analyzing OR analysis OR analyse OR analyses OR analysed OR analysing OR screen OR screens OR screened OR screening OR report OR reports OR reported OR reporting OR compare OR compares OR compared OR comparing OR comparison OR diagnose OR diagnoses OR diagnosed OR diagnosing OR diagnostic OR diagnosis OR audit OR audits OR auditing OR audited OR comply OR complies OR complied OR complying OR compliance OR validate OR validates OR validated OR validating OR validation OR Checklist OR "algorithmic hygiene" OR "algorithmic playbook" OR "algorithmic bias playbook" OR checklist OR checklists OR tool OR tools OR toolkit OR toolkits OR guideline OR guidelines OR rubric OR rubrics OR guidance OR standard OR standards OR method OR methods OR methodology OR methodologies OR checkpoint OR checkpoints OR recommendation OR recommendations OR benchmark OR benchmarks OR regulation OR regulations OR regulatory OR generalizable OR generalizability OR "prejudice remover" OR fair OR unfair OR fairness OR unfairness OR "classification parity" OR trust OR trustworthy OR trustworthiness OR PROBAST OR "Prediction Model Risk Of Bias Assessment Tool" OR "Minimum Information for Medical AI Reporting guideline" OR "Artificial Intelligence Risk Management Framework" OR "AI RMF" OR "Medical Information Mart for Intensive Care" OR MIMIC OR "balanced accuracy" OR "disparate impact" OR "equal opportunity" OR "equalized odds" OR "statistical parity") | 43,307,162 |
| 5  Race | TS=("minority group" OR ethnic OR ethnicity OR "minority groups" OR "minority population" OR "minority populations" OR "people of color" OR "person of color" OR BIPOC OR "minority health" OR race OR races OR racial OR racially OR Blacks OR "African Americans" OR "African ancestry" OR Black OR "African American" OR "Mexican Americans" OR "Hispanic or Latino" OR "Hispanic American" OR Hispanic-American OR "Hispanic Americans" OR Hispanic-Americans OR Hispanics OR Hispanic OR "Mexican American" OR Mexican-American OR "Mexican Americans" OR Mexican-Americans OR "Cuban American" OR Cuban-American OR "Cuban Americans" OR Cuban-Americans OR "Latin American" OR Latin-American OR "Latin Americans" OR Latin-Americans OR Latinos OR Latino OR Latinas OR Latina OR Latinx OR latine OR latines OR "Spanish speaking" OR Spanish-speaking OR "Spanish speakers" OR Spanish-speakers OR Mexican OR Mexicans OR "Puerto Rican" OR "Puerto Ricans" OR "Asian Americans" OR "Asian American" OR Asian OR "Japanese Americans" OR "Japanese American" OR "Chinese Americans" OR "Chinese American" OR "Vietnamese Americans" OR "Vietnamese American" OR "Asian Indian Americans" OR "Asian Indian American" OR "Cambodian Americans" OR "Cambodian American" OR "Hmong Americans" OR "Hmong American" OR "Korean Americans" OR "Korean American" OR "Filipino Americans" OR "Filipino American" OR "American Indians" OR "Alaska Natives" OR "Indigenous Peoples" OR "Indigenous People" OR "Indigenous Population" OR "Indigenous Populations" OR tribes OR "Native Peoples" OR "Native People" OR "First Nation" OR "native person" OR Alaskan OR Alaskans OR "Caribbean American" OR Caribbean-American OR "Caribbean Americans" OR Caribbean-Americans OR migrant OR migrants OR immigrant OR immigrants OR emigrant OR emigrants or demographic OR demographics OR Polynesian OR Polynesians OR "Pacific Islander" OR "Pacific Islanders" OR Hawaiian OR Hawaiians) | 1,968,002 |
| 6 | #1 AND #2 AND #3 AND #4 AND #5 | 5,173 |

4th Database: ProQuest Computer Science Database

| **Set #** | **Search Strategy** | **Results** |
| --- | --- | --- |
| 1  *Algorithms* | NOFT("Artificial Intelligence" OR "Machine Learning" OR "Deep Learning" OR Algorithm OR Algorithms OR Algorithmic OR "Artificial Intelligence" OR "augmented intelligence" OR "deep metric learning" OR "Predictive model" OR "predictive models" OR "prediction model" OR "prediction models" OR AI OR "risk score" OR "risk scores" OR "risk model" OR "risk models") | 410,974 |
| 2  Bias | NOFT("Health Status Disparities" OR "Race Factors" OR Biases OR bias OR biased OR debias OR debiased OR "de-bias" OR "de-biased" OR racism OR racial OR racist OR prejudice OR prejudices OR prejudiced OR discriminate OR discriminating OR discriminates OR discriminatory OR discriminated OR discrimination OR stereotype OR stereotypes OR stereotyped OR stereotyping OR stereotypical OR profiling OR disparity OR disparities OR inequities OR inequity OR inequality OR inequalities OR equities OR equity OR equality OR equalities OR ethics OR ethical OR ethically OR unethical OR unethically OR accountable OR accountability) | 192,745 |
| 3  Healthcare | NOFT("Health Insurance" OR "Risk Assessment" OR "Risk Management" OR "Disease Management" OR "Medical Records Systems" OR "Delivery of Health Care" OR "Health Services" OR Health OR "health care" OR healthcare OR hospitals OR hospital OR "medical center" OR "medical centers" OR "Health systems" OR "Health system" OR health-care OR medical OR clinical OR risk OR "care management" OR "disease prevention" OR Medicare OR Medicaid OR uninsured OR insured OR "Electronic Health Records" OR ((electronic OR computerized OR automated OR administrative) AND (medical or health) AND (record OR records))) | 774,748 |
| 4  Mitigation/  assessment | NOFT(mitigate OR mitigates OR mitigated OR mitigating OR mitigation OR reduce OR reduction OR reduced OR reduces OR reducing OR diminish OR diminished OR diminishing OR diminishes OR diminishment OR alleviate OR alleviates OR alleviated OR alleviating OR amend OR amends OR amended OR amending OR correct OR corrects OR corrected OR correcting OR correction OR rectify OR rectifies OR rectified OR rectifying OR rectification OR reform OR reforms OR reformed OR reforming OR reformation OR ameliorate OR strategy OR strategies OR prevent OR prevents OR preventing OR prevention OR ensure OR ensures OR ensuring OR ensured OR detect OR detects OR detected OR detecting OR detection OR limit OR limits OR limited OR limiting OR limitation OR limitations OR avoid OR avoids OR avoided OR avoiding OR remove OR removes OR removed OR removing OR removal OR decrease OR decreases OR decreased OR decreasing OR address OR addresses OR addressed OR addressing OR combat OR combats OR combated OR combatted OR combatting OR eliminate OR eliminates OR eliminated OR eliminating OR elimination OR minimize OR minimizes OR minimized OR minimizing OR minimise OR minimises OR minimised OR minimising OR Assess OR assesses OR assessed OR assessing OR assessment OR evaluate OR evaluates OR evaluated OR evaluating OR evaluation OR framework OR frameworks OR validate OR validates OR validated OR validating OR validation OR measure OR measures OR measured OR measuring OR measurement OR monitor OR monitors OR monitored OR monitoring OR analyze OR analyzes OR analyzed OR analyzing OR analysis OR analyse OR analyses OR analysed OR analysing OR screen OR screens OR screened OR screening OR report OR reports OR reported OR reporting OR compare OR compares OR compared OR comparing OR comparison OR diagnose OR diagnoses OR diagnosed OR diagnosing OR diagnostic OR diagnosis OR audit OR audits OR auditing OR audited OR comply OR complies OR complied OR complying OR compliance OR validate OR validates OR validated OR validating OR validation OR Checklist OR "algorithmic hygiene" OR "algorithmic playbook" OR "algorithmic bias playbook" OR checklist OR checklists OR tool OR tools OR toolkit OR toolkits OR guideline OR guidelines OR rubric OR rubrics OR guidance OR standard OR standards OR method OR methods OR methodology OR methodologies OR checkpoint OR checkpoints OR recommendation OR recommendations OR benchmark OR benchmarks OR regulation OR regulations OR regulatory OR generalizable OR generalizability OR "prejudice remover" OR fair OR unfair OR fairness OR unfairness OR "classification parity" OR trust OR trustworthy OR trustworthiness OR PROBAST OR "Prediction Model Risk Of Bias Assessment Tool" OR "Minimum Information for Medical AI Reporting guideline" OR "Artificial Intelligence Risk Management Framework" OR "AI RMF" OR "Medical Information Mart for Intensive Care" OR MIMIC OR "balanced accuracy" OR "disparate impact" OR "equal opportunity" OR "equalized odds" OR "statistical parity") | 4,758,704 |
| 5  Race | NOFT("minority group" OR ethnic OR ethnicity OR "minority groups" OR "minority population" OR "minority populations" OR "people of color" OR "person of color" OR BIPOC OR "minority health" OR race OR races OR racial OR racially OR Blacks OR "African Americans" OR "African ancestry" OR Black OR "African American" OR "Mexican Americans" OR "Hispanic or Latino" OR "Hispanic American" OR Hispanic-American OR "Hispanic Americans" OR Hispanic-Americans OR Hispanics OR Hispanic OR "Mexican American" OR Mexican-American OR "Mexican Americans" OR Mexican-Americans OR "Cuban American" OR Cuban-American OR "Cuban Americans" OR Cuban-Americans OR "Latin American" OR Latin-American OR "Latin Americans" OR Latin-Americans OR Latinos OR Latino OR Latinas OR Latina OR Latinx OR latine OR latines OR "Spanish speaking" OR Spanish-speaking OR "Spanish speakers" OR Spanish-speakers OR Mexican OR Mexicans OR "Puerto Rican" OR "Puerto Ricans" OR "Asian Americans" OR "Asian American" OR Asian OR "Japanese Americans" OR "Japanese American" OR "Chinese Americans" OR "Chinese American" OR "Vietnamese Americans" OR "Vietnamese American" OR "Asian Indian Americans" OR "Asian Indian American" OR "Cambodian Americans" OR "Cambodian American" OR "Hmong Americans" OR "Hmong American" OR "Korean Americans" OR "Korean American" OR "Filipino Americans" OR "Filipino American" OR "American Indians" OR "Alaska Natives" OR "Indigenous Peoples" OR "Indigenous People" OR "Indigenous Population" OR "Indigenous Populations" OR tribes OR "Native Peoples" OR "Native People" OR "First Nation" OR "native person" OR Alaskan OR Alaskans OR "Caribbean American" OR Caribbean-American OR "Caribbean Americans" OR Caribbean-Americans OR migrant OR migrants OR immigrant OR immigrants OR emigrant OR emigrants OR demographic OR demographics OR Polynesian OR Polynesians OR "Pacific Islander" OR "Pacific Islanders" OR Hawaiian OR Hawaiians) | 127,761 |
| 6 | 1 AND 2 AND 3 AND 4 AND 5 | 161 |

5th Database: ProQuest Dissertation & Theses Global

| **Set #** | **Search Strategy** | **Results** |
| --- | --- | --- |
| 1  *Algorithms* | NOFT("Artificial Intelligence" OR "Machine Learning" OR "Deep Learning" OR Algorithm OR Algorithms OR Algorithmic OR "augmented intelligence" OR "deep metric learning" OR "Predictive model" OR "predictive models" OR "prediction model" OR "prediction models" OR AI OR "risk score" OR "risk scores" OR "risk model" OR "risk models") | 274,653 |
| 2  Bias | NOFT("Health Status Disparities" OR "Race Factors" OR Biases OR bias OR biased OR debias OR debiased OR "de-bias" OR "de-biased" OR racism OR racial OR racist OR prejudice OR prejudices OR prejudiced OR discriminate OR discriminating OR discriminates OR discriminatory OR discriminated OR discrimination OR stereotype OR stereotypes OR stereotyped OR stereotyping OR stereotypical OR profiling OR disparity OR disparities OR inequities OR inequity OR inequality OR inequalities OR equities OR equity OR equality OR equalities OR ethics OR ethical OR ethically OR unethical OR unethically OR accountable OR accountability) | 363,230 |
| 3 Healthcare | NOFT("Health Insurance" OR "Risk Assessment" OR "Risk Management" OR "Disease Management" OR "Medical Records Systems" OR "Delivery of Health Care" OR "Health Services" OR Health OR "health care" OR healthcare OR hospitals OR hospital OR "medical center" OR "medical centers" OR "Health systems" OR "Health system" OR health-care OR medical OR risk OR clinical OR "care management" OR "disease prevention" OR Medicare OR Medicaid OR uninsured OR insured OR "Electronic Health Records" OR ((electronic OR computerized OR automated OR administrative) AND (medical or health) AND (record OR records))) | 989,225 |
| 4  Mitigation/  assessment | NOFT(mitigate OR mitigates OR mitigated OR mitigating OR mitigation OR reduce OR reduction OR reduced OR reduces OR reducing OR diminish OR diminished OR diminishing OR diminishes OR diminishment OR alleviate OR alleviates OR alleviated OR alleviating OR amend OR amends OR amended OR amending OR correct OR corrects OR corrected OR correcting OR correction OR rectify OR rectifies OR rectified OR rectifying OR rectification OR reform OR reforms OR reformed OR reforming OR reformation OR ameliorate OR strategy OR strategies OR prevent OR prevents OR preventing OR prevention OR ensure OR ensures OR ensuring OR ensured OR detect OR detects OR detected OR detecting OR detection OR limit OR limits OR limited OR limiting OR limitation OR limitations OR avoid OR avoids OR avoided OR avoiding OR remove OR removes OR removed OR removing OR removal OR decrease OR decreases OR decreased OR decreasing OR address OR addresses OR addressed OR addressing OR combat OR combats OR combated OR combatted OR combatting OR eliminate OR eliminates OR eliminated OR eliminating OR elimination OR minimize OR minimizes OR minimized OR minimizing OR minimise OR minimises OR minimised OR minimising OR Assess OR assesses OR assessed OR assessing OR assessment OR evaluate OR evaluates OR evaluated OR evaluating OR evaluation OR framework OR frameworks OR validate OR validates OR validated OR validating OR validation OR measure OR measures OR measured OR measuring OR measurement OR monitor OR monitors OR monitored OR monitoring OR analyze OR analyzes OR analyzed OR analyzing OR analysis OR analyse OR analyses OR analysed OR analysing OR screen OR screens OR screened OR screening OR report OR reports OR reported OR reporting OR compare OR compares OR compared OR comparing OR comparison OR diagnose OR diagnoses OR diagnosed OR diagnosing OR diagnostic OR diagnosis OR audit OR audits OR auditing OR audited OR comply OR complies OR complied OR complying OR compliance OR validate OR validates OR validated OR validating OR validation OR Checklist OR "algorithmic hygiene" OR "algorithmic playbook" OR "algorithmic bias playbook" OR checklist OR checklists OR tool OR tools OR toolkit OR toolkits OR guideline OR guidelines OR rubric OR rubrics OR guidance OR standard OR standards OR method OR methods OR methodology OR methodologies OR checkpoint OR checkpoints OR recommendation OR recommendations OR benchmark OR benchmarks OR regulation OR regulations OR regulatory OR generalizable OR generalizability OR "prejudice remover" OR fair OR unfair OR fairness OR unfairness OR "classification parity" OR trust OR trustworthy OR trustworthiness OR PROBAST OR "Prediction Model Risk Of Bias Assessment Tool" OR "Minimum Information for Medical AI Reporting guideline" OR "Artificial Intelligence Risk Management Framework" OR "AI RMF" OR "Medical Information Mart for Intensive Care" OR MIMIC OR "balanced accuracy" OR "disparate impact" OR "equal opportunity" OR "equalized odds" OR "statistical parity") | 3,783,315 |
| 5 Race/ethnicity | NOFT("minority group" OR ethnic OR ethnicity OR "minority groups" OR "minority population" OR "minority populations" OR "people of color" OR "person of color" OR BIPOC OR "minority health" OR race OR races OR racial OR racially OR Blacks OR "African Americans" OR "African ancestry" OR Black OR "African American" OR "Mexican Americans" OR "Hispanic or Latino" OR "Hispanic American" OR Hispanic-American OR "Hispanic Americans" OR Hispanic-Americans OR Hispanics OR Hispanic OR "Mexican American" OR Mexican-American OR "Mexican Americans" OR Mexican-Americans OR "Cuban American" OR Cuban-American OR "Cuban Americans" OR Cuban-Americans OR "Latin American" OR Latin-American OR "Latin Americans" OR Latin-Americans OR Latinos OR Latino OR Latinas OR Latina OR Latinx OR latine OR latines OR "Spanish speaking" OR Spanish-speaking OR "Spanish speakers" OR Spanish-speakers OR Mexican OR Mexicans OR "Puerto Rican" OR "Puerto Ricans" OR "Asian Americans" OR "Asian American" OR Asian OR "Japanese Americans" OR "Japanese American" OR "Chinese Americans" OR "Chinese American" OR "Vietnamese Americans" OR "Vietnamese American" OR "Asian Indian Americans" OR "Asian Indian American" OR "Cambodian Americans" OR "Cambodian American" OR "Hmong Americans" OR "Hmong American" OR "Korean Americans" OR "Korean American" OR "Filipino Americans" OR "Filipino American" OR "American Indians" OR "Alaska Natives" OR "Indigenous Peoples" OR "Indigenous People" OR "Indigenous Population" OR "Indigenous Populations" OR tribes OR "Native Peoples" OR "Native People" OR "First Nation" OR "native person" OR Alaskan OR Alaskans OR "Caribbean American" OR Caribbean-American OR "Caribbean Americans" OR Caribbean-Americans OR migrant OR migrants OR immigrant OR immigrants OR emigrant OR emigrants OR demographic OR demographics OR Polynesian OR Polynesians OR "Pacific Islander" OR "Pacific Islanders" OR Hawaiian OR Hawaiians) | 449,815 |
| 6 | 1 AND 2 AND 3 AND 4 AND 5 | 731 |

**Supplementary Discussion. The OPTIMIZE-AI^2^ Framework**

**The OPTIMIZE-AI^2^ Framework for Responsible AI in Healthcare Research**

The Optimizing Predictive Tools and Intelligence Models to Improve Zero Errors in AI (Accountability and Impact), or OPTIMIZE-AI**^2^** framework, is a structured guideline for building and deploying responsible AI in healthcare. The framework proposes recommendations around nine critical aspects of AI development, evaluation, and implementation in healthcare applications:

**O — Outcomes (Patient and Health system) and Values Orientation**

AI systems must demonstrate clear improvements in clinical, operational, and patient-reported outcomes. Importantly, patient-centered outcome evaluations should be stratified by race, ethnicity, gender, socioeconomic status, and other social determinants to ensure equal benefit distribution for all patients. Health system evaluations should include cost-effectiveness and return on investment (ROI) analyses to support health system performance and value-based care efforts. Evaluation should be aligned with patient-centered outcomes and system performance indicators.

**P — Performance-Driven**

High performance must be consistent across subgroups. We recommend subgroup analysis and stratified performance reporting (e.g., sensitivity, specificity, area under the curve) to detect performance disparities. AI models should be benchmarked using publicly available datasets that include representative samples of diverse populations.

**T — Transparency and Trust**

Transparency includes making information available on:

- Training data sources
- Model architecture
- Limitations and known biases
- Impact on patient groups (e.g., Black women) and patient subgroups (e.g., older Black women)

Practices such as creation of model cards, creation of datasheets for datasets, and documentation of fairness evaluations can promote end-user trust.

**I — Inclusivity by Design**

Representative data inclusion is critical. Developers should also:

- Conduct equity impact assessments
- Use community-engaged methods to incorporate lived experience
- Apply participatory design principles to prioritize usability across patient groups

**M — Mitigation of Bias**

Bias mitigation should be addressed at multiple levels: pre-processing (e.g., reweighting), in-processing (e.g., adversarial debiasing), and post-processing (e.g., equalized odds calibration). Based on our review:

- Pre-processing methods (e.g., reweighting, sampling) are often more interpretable and applicable in early-stage development.
- In-processing techniques (e.g., adversarial training) may be more effective in minimizing model bias but require deeper model access and expertise.
- Post-processing methods are easier to apply when working with black-box models but may trade off accuracy for fairness.

No single mitigation method is universally superior; rather, effectiveness depends on the specific context, including data quality, algorithmic complexity, and clinical application. We recommend using multiple fairness metrics—such as demographic parity, equal opportunity, equalized odds, and calibration across groups—to evaluate trade-offs and select appropriate strategies.

**I — Integration into Workflow**

AI tools should be co-designed with end-users to fit into clinical workflows. Usability testing should assess whether AI tools enhance or burden clinical decision-making. Workflow integration should also consider alert fatigue, decision autonomy, and team-based care models.

**Z — Zero Harm Goal**

A “do no harm” approach must guide the development and deployment process, particularly for “high-risk” tools affecting diagnosis or treatment decisions. Safety reviews—simulating model impact across patient groups and subgroups—should be a routine part of validation.

**E — Evaluation and Monitoring**

Post-deployment evaluation should be ongoing and include:

- Real-time audit trails
- Performance drift monitoring
- Fairness dashboards
- Patient and clinician feedback loops Tools should be re-evaluated when deployed in new settings, populations, or electronic health record systems to ensure continued fairness and effectiveness.

**AI^2^ — Artificial Intelligence (with Accountability and Impact)**

Accountability requires governance frameworks, cross-disciplinary oversight, and mechanisms for redress when harm occurs. Alignment with regulatory guidance (e.g., FDA, NIST, ONC), as well as internal algorithm governance boards, is key to ensuring ethical and impactful use of AI.

**Supplementary Table 1. Quality Assessment Criteria for Quantitative Studies**

| **Author** | **Quality Assessment Criteria for Quantitative Studies** | | | | | | | | | | | | | | **SUMMARY SCORE** |
| --- | --- | --- | --- | --- | --- | --- | --- | --- | --- | --- | --- | --- | --- | --- | --- |
|  | **Question / objective sufficiently described?** | **Study design evident and appropriate?** | **Method of subject/comparison group selection *or* source of information/input variables described and appropriate?** | **Subject (and comparison group, if applicable) characteristics sufficiently described?** | **If interventional and random allocation was possible, was it described?** | **If interventional and blinding of investigators was possible, was it reported?** | **If interventional and blinding of subjects was possible, was it reported?** | **Outcome and (if applicable) exposure measure(s) well defined and robust to measurement / misclassification bias?** | **Sample size appropriate?** | **Analytic methods described/justified and appropriate?** | **Some estimate of variance is reported for the main results?** | **Controlled for confounding?** | **Results reported in sufficient detail?** | **Conclusions supported by the results?** |  |
| Adeli (2021) | YES | PARTIAL | YES | YES | NA | NA | NA | YES | YES | YES | YES | YES | YES | YES | 0.95 |
| Afrose (2021) | YES | YES | YES | YES | NA | NA | NA | YES | YES | YES | YES | NO | YES | YES | 0.91 |
| Akbilgic (2018) | YES | YES | YES | YES | NA | NA | NA | PARTIAL | PARTIAL | YES | YES | YES | YES | YES | 0.91 |
| Allen (2020) | YES | YES | YES | YES | NA | NA | NA | YES | YES | YES | YES | YES | YES | YES | 1.00 |
| Barda (2021) | YES | YES | YES | YES | NA | NA | NA | YES | NA | YES | YES | YES | YES | YES | 1.00 |
| Borgese (2021) | YES | YES | YES | YES | NA | NA | NA | YES | YES | YES | YES | PARTIAL | YES | YES | 0.95 |
| Buckley (2022) | YES | YES | YES | YES | NA | NA | NA | YES | YES | YES | YES | YES | YES | YES | 1.00 |
| Burlina (2021) | YES | YES | PARTIAL | YES | NA | NA | NA | YES | NO | PARTIAL | YES | YES | YES | YES | 0.82 |
| Do (2022) | YES | YES | YES | PARTIAL | NA | NA | NA | YES | YES | YES | NO | NO | YES | YES | 0.77 |
| Foryciarz (2022) | YES | YES | YES | YES | NA | NA | NA | YES | PARTIAL | YES | YES | YES | YES | YES | 0.95 |
| Gama (2021) | YES | YES | YES | YES | NA | NA | NA | YES | NO | YES | YES | YES | YES | YES | 0.91 |
| Gao (2020) | YES | YES | YES | PARTIAL | NA | NA | NA | YES | NA | YES | YES | YES | YES | YES | 0.95 |
| Gianattasio (2020) | YES | YES | YES | YES | NA | NA | NA | YES | YES | YES | YES | YES | YES | YES | 1.00 |
| Hammond (2020) | YES | YES | YES | YES | NA | NA | NA | YES | NO | YES | NO | YES | YES | YES | 0.82 |
| Howard (2021) | YES | YES | YES | YES | NA | NA | NA | YES | YES | YES | PARTIAL | NA | YES | YES | 0.95 |
| Huang (2022) | YES | YES | YES | YES | NA | NA | NA | YES | NA | YES | YES | NO | YES | YES | 0.90 |
| Joo (2022) | YES | YES | YES | YES | NA | NA | NA | YES | NO | YES | YES | NA | YES | YES | 0.90 |
| Landy (2021) | YES | YES | YES | YES | NA | NA | NA | PARTIAL | YES | YES | NO | YES | YES | YES | 0.86 |
| Lin (2022) | YES | YES | YES | YES | NA | NA | NA | YES | YES | YES | YES | NO | YES | YES | 0.91 |
| Mosteiro (2022) | YES | YES | YES | YES | NA | NA | NA | YES | YES | YES | YES | NO | YES | YES | 0.91 |
| Noseworthy (2020) | YES | YES | YES | YES | NA | NA | NA | YES | YES | YES | NO | NO | YES | YES | 0.82 |
| Obermeyer (2019) | YES | YES | YES | YES | NA | NA | NA | YES | YES | YES | YES | YES | YES | YES | 1.00 |
| Park (2022) | YES | YES | YES | YES | NA | NA | NA | YES | NO | YES | YES | NO | YES | YES | 0.82 |
| Park (2021) | YES | YES | YES | YES | NA | NA | NA | YES | YES | YES | YES | YES | YES | YES | 1.00 |
| Perez Alday (2022) | YES | YES | YES | YES | NA | NA | NA | PARTIAL | YES | YES | NO | NO | PARTIAL | YES | 0.73 |
| Pfohl (2021) | PARTIAL | YES | YES | YES | NA | NA | NA | PARTIAL | YES | YES | YES | PARTIAL | YES | YES | 0.86 |
| Pierson (2021) | YES | YES | YES | YES | NA | NA | NA | YES | YES | YES | YES | YES | YES | YES | 1.00 |
| Puyol-Anton (2021) | YES | YES | YES | NO | NA | NA | NA | YES | YES | YES | NO | NO | PARTIAL | PARTIAL | 0.64 |
| Radovanović (2019) | YES | YES | YES | NO | NA | NA | NA | YES | YES | YES | NO | NO | PARTIAL | PARTIAL | 0.64 |
| Reeves (2022) | YES | PARTIAL | YES | YES | NA | NA | NA | NA | YES | YES | YES | NO | YES | YES | 0.85 |
| Segar (2022) | YES | YES | YES | YES | NA | NA | NA | NA | YES | YES | YES | YES | YES | YES | 1.00 |
| Segar (2021) | YES | YES | YES | YES | NA | NA | NA | NA | YES | YES | YES | YES | YES | YES | 1.00 |
| Thompson (2021) | YES | YES | YES | YES | NA | NA | NA | NA | YES | YES | YES | YES | YES | YES | 1.00 |
| Toseef (2022) | YES | YES | YES | PARTIAL | NA | NA | NA | NA | YES | YES | YES | NO | YES | YES | 0.85 |
| Weissman (2021) | YES | YES | YES | YES | NA | NA | NA | NA | YES | YES | YES | YES | YES | YES | 1.00 |

**Supplementary Table 2. Summary of the Included Studies**

| **Author & Year** | **Country** | **Clinical Category** | **Bias Type** | **Sample** | **Model Description** | **Outcome Measures** | **Results** |
| --- | --- | --- | --- | --- | --- | --- | --- |
| Adeli (2021) | United States | Medical Imaging/ Radiology, HIV: Head MRI in HIV, Facial Image Recognition | Age, Gender, Race/Ethnicity/Skin Color | For each of 3 trials:  1) Synthetic dataset - 512 control and 512 experimental images  2) HIV diagnosis based on brain MRIs - 223 control and 122 HIV patients  3) Gender prediction based on facial image - 561 female and 692 male subjects | Bias-resilient neural network (BR-Net), based on adversarial training strategies | 3 trials:  1) Accuracy in relation to use of protected variables 2) Brain MRi classification task performance (HIV vs Healthy/Control) with age as protected feature  3) Facial image classification task performance (male vs female gender) with skin shade/ color as protected feature | BR-Net consistently performed better than baseline models while minimizing bias with respect to protected variables. |
| Afrose (2021) | United States | Clinical Operations, Oncology: Mortality/Decompensation, Cancer Survival | Age, Dataset, Race/Ethnicity/Skin Color | For each of 3 tasks:  1) In-hospital mortality and decompensation clinical prediction - 14,681 time-series samples from MIMIC III dataset 2) 5-year breast cancer survival prediction - training set of 199,000 samples from SEER dataset 3) 5-year lung cancer survival prediction - training set of 164,443 samples from SEER dataset | Double prioritization (DP) and prioritized reweighting | Primary: Performance of algorithms with and without bias correction for in-hospital mortality and decompensation prediction (IHM), 5-year breast cancer survival (BCS), and 5-year lung cancer survival (LCS)  Secondary: Fairness performance | DP effectively boosted the recall of the minority class for underrepresented patient groups and reduced the disparity among age and race groups.   DP exhibited the smallest racial and age disparities among the tested methods. |
| Akbilgic (2018) | United States | Surgery, Pediatrics: Pediatric Post-Operative Mortality | Race/Ethnicity/Skin Color | NSQIP Pediatric Participant Use File (PUF) Training get: Years 2012-2014 data containing 183,233 surgical cases  Validation set: Year 2015 data containing 84,056 surgical cases | Classification tree done with the χ2 automatic interaction detector algorithm | Primary: D30 (death within 30 days of surgery) Secondary: Risk factors for D30 | Race-specific classification tree models performed better than those that were not race-specific |
| Allen (2020) | United States | Clinical Operations: Mortality | Race/Ethnicity/Skin Color | MIMIC III dataset from 2001-2012, narrowed to 28,460 patients | XGBoost model, a gradient-boosting technique that combines multiple decision trees | Primary: In-hospital mortality prediction Secondary: Fairness performance | The model outperformed all rules-based comparator scoring systems in predicting in-hospital mortality. |
| Barda (2021) | Israel | Cardiology/ Endocrinology: Cardiovascular Events (PCE), Osteoporotic Fractures (FRAX) | Any underrepresented subpopulation | Data from Clalit Health Services (CHS):  PCE population of 1,021,041 patients;  FRAX population of 1,116,324 patients | An adapted version of the fairness algorithm designed by Hebert-Johnson et al. 2017 | Primary: Calibration and discrimination performance of PCE and FRAX before/after deploying fairness algorithm | The fairness algorithm improved subpopulation calibration. It could be applied to predictions generated in any manner given a training set of patients with an adequate follow-up period and a minimum subpopulation size. |
| Borgese (2021) | United States | Behavioral Health: Alcohol Use Disorder | Race/Ethnicity/Skin Color | Convenience sample of two cohorts: trauma patient encounters in the emergency department (n=1326) and an independent group of hospitalized patients in the medical/surgical wards (n=999). | NLP classifier from their previous work | Primary: Improvement in bias metrics from baseline | In trauma patients, the baseline NLP classifier underpredicted unhealthy EtOH use for Hispanic vs NHW participants, and it was less accurate for those aged <44 and males. After re-calibration, in the Hispanic subgroup, accuracy (scaled Brier score) improved most with combined intercept+slope re-estimation, but underestimation persisted.  In non-trauma inpatients, baseline NLP classifier underpredicted unhealthy EtOH use for those aged <44 and NH-Black participants vs NHW participants. After re-calibration, FNR decreased for all approaches vs baseline, but minimal improvement in the other metrics was observed. Accuracy (scaled Brier scores) did not improve vs baseline for any approach. |
| Buckley (2022) | United States | Obstetrics: TOLAC/VBAC | Race/Ethnicity/Skin Color | 1241 pregnant patients | Multivariate logistic regression model to model TOLAC success using predictors from Groabman et al. 2021 | Primary: Compare the predictive ability of the VBAC calculator in predicting the success of TOLAC with and without the inclusion of race and ethnicity variables. Secondary: Determine whether or not race or ethnicity was associated with an increased risk of morbidity | There was no difference in model performance when race/ethnicity was removed. Race/ethnicity was associated with increased morbidity (overall complication rate, PPH, abruption, IAI) |
| Burlina (2021) | United States | Endocrinology, Ophthalmology: Diabetic Retinopathy | Race/Ethnicity/Skin Color | Retinal images from two subgroups (individuals with lighter-skin and darker-skin) in a dataset that included 88,692 fundi and 44,346 individuals. | Standard deep learning algorithm, trained using transfer learning (originally from ResNet50), to predict diabetic retinopathy, comparing two debiasing methods | Primary: Determine whether the proposed generative models could debias the diagnostic AI. Secondary: Assess the performance of the debiased DLSs | The baseline AI system showed a significant disparity in accuracy between subpopulations. However, the use of novel generative methods for addressing missing subpopulation training data resulted in closer parity in accuracy across subpopulations. |
| Do (2022) | United States | Infectious Disease: COVID-19 | Age | Patients of age 50+ with laboratory-confirmed COVID-19 | Logistic regression for COVID-19 mortality prediction | COVID-19 mortality | A JFM approach was applied to logistic regression models to estimate group-specific classifiers using an objective function that incorporated fairness criteria for prediction. The proposed JFM algorithm outperformed a group-separate model in all age groups (<50 yo) as well as a group-ignorant model, in age groups > 65 yo. |
| Foryciarz (2022) | United States | Cardiology: Atherosclerotic Cardiovascular Disease (ASCVD) Risk | Race/Ethnicity/Skin Color | Individuals aged 40–79 who did not present a history of myocardial infarction, stroke, coronary bypass surgery, angioplasty, congestive heart failure or atrial fibrillation or who were receiving statins at the time of the initial examination. | Unconstrained, group-recalibrated, and equalized odds-constrained versions of the 10-year ASCVD risk estimators (logistics regression) | 10-year ASCVD risk | Compared with the unconstrained model, group-recalibration improved calibration in the groups at guideline-concordant therapeutic thresholds. However, it increased intergroup differences in error rates (FPR and FNR). |
| Gama (2021) | United Kingdom | Nephrology: Chronic Kidney Disease | Race/Ethnicity/Skin Color | Black adults over 18 years of age with a Cr-EDTA (chromium-51 labeled ethylenediamine tetraacetic acid) mGFR study. Participants with albumin <30g/l, liver disease, <18 years, of non-Black or non-White self-reported ethnicity were excluded. | Estimated glomerular filtration rate equations | Estimated Glomerular Filtration Rate (eGFR) | Removal of race-correction from eGFR calculations improves GFR estimation in Black patients |
| Gao (2020) | United States | Oncology: Prognosis | Dataset, Race/Ethnicity/Skin Color | Undefined. Authors used cancer omics data from two data sources: The Cancer Genome Atlas (TCGA) cohort and the Multiple Myeloma Research Foundation (MMRF) CoMMpass cohort. | Deep neural network with transfer learning and a logistic regression in the output layer | Four clinical outcome endpoints: overall survival (OS), disease-specific survival (DSS), progression-free interval (PFI), and disease-free interval (DFI) | Transfer learning produced models with significantly better performance for the African American group compared to the models from mixture learning. |
| Gianattasio (2020) | United States | Neurology: Dementia | Race/Ethnicity/Skin Color | Participants in the Health and Retirement Study - HRS (>50 y/o) and the Aging, Demographics, and Memory Study - ADAMS (>70 y/o) studies. | Logistic regression and a super learner two-stage ensemble algorithm learn a best suited ML algorithm from a family of ML algorithms (decision trees, Boosting, basso, elasticNet, kNN, SVM). Three algorithms were identified: The Hurd model, an expert model, and a LASSO model | Dementia status | The algorithms developed reduced differences in performance between racial/ethnic groups: They achieved ≤3 percentage point difference in sensitivity and ≤5 percentage point difference in specificity across racial/ethnic groups.  Popular machine-learning models were not superior to traditional regression-based models when the sample size and predictor set was relatively small. |
| Hammond (2020) | United States | Clinical Operations: Hospitalization, Mortality | Race/Ethnicity/Skin Color | Nationally representative sample of US Medicare beneficiaries | Logistic and linear regressions examining the relationship between race/ethnicity and annual costs of care, all-cause hospitalization, cardiovascular hospitalization, and death | 4 outcomes: -all-cause hospitalization -cardiovascular hospitalization -death -annual cost | Including social risk in models predicting costs and clinical outcomes improved model accuracy among racial and ethnic minorities. |
| Howard (2021) | United States | Oncology, Genomics: Prognosis, Gene Expression/Mutations | Age, BMI, Gender, Race/Ethnicity/Skin Color | Cancer cohorts from the Cancer Genome Atlas (TCGA) | Convolutional neural network models | Lab sites and demographic features (real outcomes: progression-free survival, gene expression, genetic mutations) | The distribution of outcomes of interest across sites should be carefully examined. A submitting site should be isolated to either the cohort used for training or for testing a model. A quadratic programming approach can maintain optimal stratification while still isolating submitting sites to either training or validation datasets. |
| Huang (2022) | United States | Cardiology, Nephrology: AKI after PCI | Race/Ethnicity/Skin Color | Patients from the National Cardiovascular Data Registry (NCDR) CathPCI Registry with an AKI rate of 7.4% | eGFR was used as predictor in multiple models. A series of logistic-regression based models and ML models were trained by changing how eGFR was calculated, including a model with race correction factor included in the eGFR calculation (Model 1), a model with the race correction factor excluded (Model 2) and a model including race as an additional and independent predictor (Model 3) | Post-percutaneous coronary intervention Acute Kidney Injury | Removing the race correction in eGFR calculation had a positive effect on reducing the underestimation of the risk of AKI following PCI for Black patients. Adding race as a covariate improved calibration in both logistic-regression based model and ML (XGBoost) model. |
| Joo (2022) | Republic of Korea | Nephrology: Immunoglobin A Nephropathy | Race/Ethnicity/Skin Color | Adult patients (>= 18 yo) with immunoglobin A nephropathy | The International IgA Nephropathy Prediction tool | 50% decline in estimated glomerular filtration rate (eGFR) from the time of biopsy or end-stage kidney disease | The updated eGFR model with the Korean coefficient showed acceptable calibration (good agreement between predicted risk and observed outcome), suggesting that race/ethnicity factors should be considered when constructing prediction models. |
| Landy (2021) | United States | Pulmonology, Oncology: Lung Cancer Screening | Race/Ethnicity/Skin Color | Individuals from the US 2015 National Health Interview Survey (NHIS) aged 50-80 with any history of smoking   USPSTF-2013: Total 8,024,510  USPSTF-2020: Total 14,508,450 | The LYFS-CT model to predict individualized life-years gained from National Lung Screening Trial (NLST)-like screening | Primary: The number and proportion of US individuals aged 50-80 years who ever smoked who were eligible for screening, sensitivity for preventable lung-cancer deaths, and screening efficiency. Secondary: The sensitivity and efficiency per 10 life-years gained | 1) The updated 2020 USPSTF lung cancer screening guidelines expanded the eligible population for screening, which partially reduced racial disparities. 2) The USPSTF-2020 guidelines unintentionally widened the relative disparities between Whites and minority groups. 3) However, when the USPSTF-2020 guidelines were combined with the LYFS-CT model's method of selecting high-benefit individuals, the disparities for African Americans were nearly eradicated |
| Lin (2022) | United States | Obstetrics: Preeclampsia/Eclampsia | Dataset, Race/Ethnicity/Skin Color | 1758 participants across 8 clinics | PEPrML (PreEclampsia Predictor with Machine Learning), based on fandom forest architecture | Primary: prediction of preeclampsia with severe features or eclampsia at different time points during pregnancy Secondary: n/a | The model could be used as a screening tool as early as 6-13 weeks of gestation to help clinicians identify participants who may subsequently develop preecalmpsia, confirming suspected cases or identifying unsuspected cases. However, they generally performed better when used later in pregnancy. |
| Mosteiro (2022) | Netherlands | Behavioral Health: Benzodiazepine Use | Gender | Adult psychiatric patients | Logistic regression, Random Forest | future administrations of benzodiazepines | Models trained to predict future administrations of benzodiazepines based on past data were biased by patients’ gender. Reweighing the data (a preprocessing step) seemed to significantly mitigate this bias, without loss of performance. The in-processing method with a prejudice remover also mitigated this bias, but at a cost to performance. |
| Noseworthy (2020) | United States | Cardiology: Left Ventricular Ejection Fraction | Race/Ethnicity/Skin Color | Adult patients (>18 yo) with an ECG and a standard transthoracic echocardiogram | CNN | patients with a left ventricular ejection fraction ≤35% from the 12-lead ECG | Convolutional neural networks predicted low left ventricular ejection fraction from the ECG despite variation of ECG characteristics by race. Reporting of performance among diverse ethnic, racial, age, and sex groups for all new artificial intelligence tools is recommended to ensure responsible use of artificial intelligence in medicine |
| Obermeyer (2019) | United States | Clinical Operations: Complex Care Management | Race/Ethnicity/Skin Color | Primary care patients enrolled in risk-based contracts from 2013 – 2015, self-identifying as Black (n=6079) or White (n=43,539) | Live, scaled algorithm deployed nationwide to target patients for high-risk care management programs | Determine mechanism for algorithm bias | The degree of observed bias from a prediction model may be highly dependent on the labels used to train it. |
| Park (2022) | United States | Behavioral Health: Mental Health | Gender | The data set from a previous study by Singh and Long | Five popular classification algorithms, including logistic regression, support vector machine, random forest, k-nearest neighbors, and multilayer perceptron neural networks using the scikit-learn library | Determine whether it is possible to reduce the level of bias while maintaining high accuracy | There was a statistically significant reduction in algorithm performance disparity after the application of the DIR approach by adapting the data used for modeling. |
| Park (2021) | United States | Behavioral Health: Postpartum Depression (PPD) | Dataset | Females with live birth record and enrolled in Medicaid | Risk prediction algorithm for postpartum ML models (logistic regression, random forest, extreme gradient boosting) | Postpartum depression and mental health service use | Application of a reweighing method was associated with a greater reduction in algorithmic bias for postpartum depression and mental health service utilization prediction between White and Black individuals. |
| Perez Alday (2022) | Multiple countries | Cardiology: Arrhythmias | Age, Gender, Race/Ethnicity/Skin Color | The 2021 PhysioNet Challenge included a total of 131,155 ECG recordings from 9 databases, 4 countries, and 3 continents | A LASSO model using 11 algorithms ranked based on forward selection on the training set was used with the reduced bias model | Performance differences in algorithms across sex, race, and age groups | a. Sex analysis: Recording-wise accuracies were higher, and the challenge score was lower for female ECGs. b. Age analysis: Performance varied significantly with age, particularly showing a drop in performance for individuals aged 40–49 years old. Recording-wise accuracy decreased with each decade c. Race analysis: Recording-wise accuracy was lower for Black participants and higher for Asian participants. |
| Pfohl (2021) | United States | Clinical Operations: 30-day Hospital Readmission | Age, Gender, Race/Ethnicity/Skin Color | Admissions from 198,644 patients in STARR, 8,074,571 patients in Optum CDM, and 26,170 patients in MIMIC-III. | LBFGS algorithm implemented in scikit-learn | 30-day hospital readmission | Without fairness-promoting regularization, there were substantial differences in group-level model performance measures such as AUROC, average precision, and cross entropy loss. |
| Pierson (2021) | United States | Pain Management: Osteoarthritis/Pain Management | BMI, Education, Gender, Income, Race/Ethnicity/Skin Color | 4,172 patients in the United States who had or were at high risk of developing knee osteoarthritis. | Algorithmic pain prediction (ALG-P), convolutional neural network | The predictive performance of the algorithm in reducing disparities | The study found that the algorithmic measure of osteoarthritis severity (ALG-P) achieved better predictive performance for pain than baseline (Kellgren–Lawrence grade [KLG]), even within racial and socioeconomic subgroups. Models trained under both diverse and non-diverse conditions outperformed KLG. |
| Puyol-Anton (2021) | United Kingdom | Cardiology: Cardiac MRI | Race/Ethnicity/Skin Color | 5,903 subjects from the UK Biobank dataset | nnU-Net model | The effectiveness of different bias mitigation strategies on reducing racial bias in the models | All three bias mitigation strategies (i.e., S\stratified batch sampling, fair meta-learning for segmentation, and protected group models) were effective in reducing racial bias. |
| Radovanović (2019) | United States | Clinical Operations, Pediatrics: Pediatric 30-day Hospital Readmission | Gender | Pediatric hospital readmission data containing 66,994 hospital admissions and 851 diagnoses. | Logistic regression model | 30-day hospital readmission | The reweighting strategy maintained similar predictive performance while improving fairness. The adversarial network showed decreased performance but improved fairness. The equality of odds technique optimized fairness metrics but at the cost of other performance measures. |
| Reeves (2022) | United States | Behavioral Health: Suicide | Class, Race/Ethnicity/Skin Color | Emergency department patients who were 5 years or older, had a California residential zip code, and had fewer than 500 emergency department visits | Four predictive model methods: logistic regression, naive Bayes, gradient boosting (XGBoost) and random forests | Suicidal death | Building separate models for different racial/ ethnic groups or using the equity method on the training set decreased the range in performance. |
| Segar (2022) | United States | Cardiology: Congestive Heart Failure | Race/Ethnicity/Skin Color, SDOH | Training data set that included 123,634 hospitalized patients with HF who were enrolled in the GWTG-HF registry. For the present analysis, 677, 140 patients from 634 hospitals between January 1, 2010, and December 31, 2020, were considered for model development and validation | Random forest-based ML approaches | In-Hospital Mortality in Patients With Heart Failure | ML models for HF mortality demonstrated superior performance to the traditional and rederived logistic regressions models using race as a covariate. The addition of SDOH parameters improved the prognostic utility of prediction models in Black patients but not non-Black patients. |
| Segar (2021) | United States | Cardiology: Congestive Heart Failure | Race/Ethnicity/Skin Color | The derivation cohort for Black and White adults consisted of 4141 participants from JHS and 7858 participants from ARIC, respectively (Supplemental Figure I). MESA and the DHS contributed 1848 and 973 participants to the external validation cohort for Black adults (n=2821), respectively, while ARIC contributed 1024 participants. | Six candidate ML-based and traditional Cox algorithms: (1) oblique random survival forest (oRSF), (2) forward stepwise Cox regression, (3) LASSO Cox regression, (4) ridge Cox regression, (5) boosted Cox regression, and (6) gradient boosted trees | Model performance, by racial group - predicting 10-year risk of incident HF | Race-specific and ML-based HF risk models that integrated clinical, laboratory, and biomarker data demonstrated superior performance compared with traditional HF risk and non–race-specific ML models. |
| Thompson (2021) | United States | Behavioral Health: Opioid Use Disorder | Race/Ethnicity/Skin Color | Local EHR data: unplanned adult inpatient encounters (n = 53 974) | A previously developed CNN opioid classifier from Sharma et al. 2020 | Prediction of opioid misuse | Posthoc recalibrations eliminated bias in FNR with minimal changes in other subgroup error metrics. The Black FNR subgroup had higher risk scores for readmission and mortality than the White FNR subgroup and a higher mortality risk score than the Black true positive subgroup (P < .05). |
| Toseef (2022) | United States | Oncology: Prognosis | Race/Ethnicity/Skin Color | Cancer genome research project data, including TCGA, TARGET and MMRF CoMMpass, incorporating varying numbers of protein and mRNA features from patients of differing ancestry | Deep neural network model trained with public data, following the same schemes as Gao & Cui (2020) | Cancer prognosis defined through 4 endpoint outcomes: 1) disease-specific survival (DSS), 2) the overall survival of the patient (OS), 3) progression-free interval (PFI) and 4) disease-free interval (DFI) | The model improved the transfer learning scheme of multiethnic experiments with promising AUROC. |
| Weissman (2021) | United States | Clinical Operations, Cardiology: CHF 30-day Hospital Readmission | Race/Ethnicity/Skin Color | 1,316 patients discharged with a primary diagnosis of congestive heart failure (CHF) from the University of Pennsylvania Health System, 2015-2017 | Two regression models based on clinical and demographic variables - one with the ADI as a predictor variable, and one without | 30-day hospital readmission | Inclusion of the ADI did not significantly improve model performance or algorithmic equity. |

Abbreviations: ADI, area deprivation index; AKI, acute kidney injury; ARIC, Atherosclerosis Risk in Communities Study; ASCVD, atherosclerotic cardiovascular disease; AUROC, area under the receiving operator characteristic curve; BMI, body mass index; COVID-19, coronavirus disease 2019; CNN, convolutional neural network; DHS, Dallas Heart Study; DLSs, diagnostic laboratory services; ECG, electrocardiography; DIR, Developmental, Individual-differences, and Relationship-based model; eGFR, estimated glomerular filtration rate; EHR, electronic health record; FNR, false negative rate; FPR, false positive rate; FRAX; GWTG-HF, Get With The Guidelines – Heart Failure Registry; HF, heart failure; HIV, human immunodeficiency virus; IAI, intraamniotic infection; JFM, joint fairness modelling; JHS, Jackson Heart Study; KLG, Kellgren–Lawrence grade; kNN, k-nearest neighbors; LASSO, least absolute shrinkage and selection operator; LBFGS, limited-memory Broyden–Fletcher–Goldfarb–Shanno; MESA, Multi-Ethnic Study of Atherosclerosis; mGFR, measured glomerular filtration rate; MIMIC III, Medical Information Mart for Intensive Care III; ML, machine learning; MMRF, Multiple Myeloma Research Foundation; MRI, magnetic resonance imaging; NHW, non-Hispanic White; NLP, natural language processing; NSQIP, National Surgical Quality Improvement Program; PCE, pooled cohort equation; PCI, percutaneous coronary intervention; PPH, postpartum hemorrhage; SDOH, social determinant of health; SEER, Surveillance, Epidemiology, and End Results; SVM, support vector machine; TARGET, Therapeutically Applicable Research to Generate Effective Treatments; TCGA, The Cancer Genome Atlas Program; TOLAC/VBAC, trial of labor after cesarean/vaginal birth after cesarean; UK, United Kingdom; US, United States; USPSTF, United States Preventive Services Task Force.

**Supplementary Table 3. Approaches to Identifying and Mitigating Bias in Clinical Algorithms**

| **Author** | **Algorithm Stage of Bias Mitigation Strategy** | | | | **Bias Mitigation Strategy Reported** | **Fairness Metric** | **Effective in Reducing Bias?** | **Results in Reducing Bias** |
| --- | --- | --- | --- | --- | --- | --- | --- | --- |
|  | **Algorithm Design** | **Pre-Processing** | **In-Processing** | **Post-Processing** |  |  |  |  |
| Adeli (2021) |  |  | X |  | Introduction of a new adversarial loss function to minimize bias from protected variables (e.g. age and skin shade) | All: squared distance correlation (dcor2) and mutual information; In experiment (3): In GS-PPB, Equality of Opportunity (EO) | Y | The developed algorithm, BR-Net, was very effective at generating accurate results without depending on protected variables (age, skin color). |
| Afrose (2021) | X | X | X |  | Two techniques: 1) double prioritization, a form of oversampling; 2) prioritized reweighting, a method that adds weight to minority sub-groups | Relative disparity metric | Y | The double prioritized bias correction technique effectively boosted the recall of the minority class for underrepresented patient groups and reduced the disparity among age and race groups. It exhibited the smallest racial and age disparities among the tested methods. |
| Akbilgic (2018) | X |  | x |  | Race-specific risk classification modeling using classification tree | Sensitivity, specificity | Y | Race-specific classification tree models performed better than those that were not race-specific. They also identified different risk factors for Black patients and greater prevalence of some of them. |
| Allen (2020) |  | X |  |  | A two-step pre-processing technique: 1) separate patients into age groups (e.g., age <18, 18-29, 30-39, etc); 2) reweight individual training examples based on the ratio of the expected vs observed combination of race and mortality status (i.e., a demographic prevalence ratio, previously described by Kamarin and Calders) | Equal opportunity difference | Y | The XGBoost algorithm trained on pre-processed data was found to be unbiased. Comparing the XGBoost model trained on preprocessed vs non-preprocessed training data, preprocessing resulted in lower bias and similar feature importance for all measured features across racial groups. |
| Barda (2021) |  |  |  | X | A postprocessing fairness algorithm that improves subpopulation calibration | Calibration as measured by CITL (calibration in the large) and CS (calibration scope) | Y | (1) The average CITL and CS across subpopulations improved. In addition, the variance in calibration between subpopulations was greatly reduced. AUROC, AUPRC, and Brier Scores were all unaffected by fairness algorithm implementation.  (2) Training a model for each nonoverlapping subpopulation is not a viable solution to the problem of subpopulation miscalibration due to large eventual variance of calibration metrics. |
| Borgese (2021) | X |  |  | x | Recalibration (via intercept vs slope vs combined intercept+slope) by subgroup | Calibration, false dicovery rate (FDR), false positive rate (FPR), false omission rate (FOR), false negative rate (FNR) | N | The authors detected bias in their NLP classifier and were unable to adequately address this bias through post hoc recalibration methods. |
| Buckley (2022) | X |  | x |  | Race/ethnicity removal | AUROC | N/A | Removing race/ethnicity from the multivariate ML model had no effect on predictive accuracy of TOLAC. |
| Burlina (2021) |  | X |  |  | Augmentation of training image dataset -- their two methods involve starting with real images and accentuating features vs starting with synthetic images and doing the same. | Prediction Accuracy parity (delta in accuracy) | Y | Relative to the baseline model, the models trained on debiased datasets ("retina appearance-optimized" and "DR-optimized") demonstrate improved metrics: accuracy; sensitivity; Specificity; and AUROC. |
| Do (2022) |  |  | x |  | A JFM that incorporates fairness criteria for prediction. | None. Fairness is evaluated through estimation of prediction performance across (age) groups | y | The JFM performed better across all age groups than the separate model did. |
| Foryciarz (2022) |  |  |  | x | Local recalibration at guideline-concordant therapeutic thresholds | - Calibration at the aggregate level (ACE) and at the local level (TCE). - IGSD between the four group-specific values of ACE, TCE, and FNR. IGSD captures the degree of performance disparity between groups; high IGSD in FPR and FNR corresponds to an equalized odds* disparity, and high IGSD in TCE corresponds to a treatment rule disparity. | y | Recalibrating the model separately for each group increased compatibility with guidelines at low levels of risk, while increasing intergroup differences in error rates. |
| Gama (2021) |  |  | x |  | Removal of ethnicity adjustment eGFR equations (eGFR-MDRD and eGFR-CKD-EPI) | Assessment of performance before and after ethnicity adjustment | y | Removal of race-correction from eGFR calculations improved GFR estimation in Black patients. |
| Gao (2020) |  |  | x |  | Transfer learning for improving machine learning model performance for data-disadvantaged ethnic groups. | Evaluation of performance improvement, defined by the performance disparity gap, G: G = mean(AUROC_EA) - mean(AUROC_AA) *EA: European Americans; AA: African Americans | y | Transfer learning produced models with significantly better performance for the African American group compared to the models from mixture learning. |
| Gianattasio (2020) |  |  |  | x | Variation of the model probability threshold and selection of the threshold that meets a set of performance criteria that guarantees fairness between groups | Minimization of cross-group differences in sensitivity and specificity | y | There were reduced differences in performance between racial/ethnic groups. |
| Hammond (2020) |  |  | x |  | Adjustment of SDOH in risk models | Fairness was indirectly evaluated through measurement of improvement in performance in minority group after including SDOH predictors. | y | Including social risk in models predicting costs and clinical outcomes improved model accuracy among racial and ethnic minorities. |
| Howard (2021) |  |  | x |  | Covex optimization/Quadratic programming to isolate individual lab sites to individual folds for cross-validation | Model performance for preserved-site cross validation in TCGA across multiple cancer types | y | A quadratic programming approach can maintain optimal stratification while still isolating submitting sites to either training or validation datasets. Quadratic programming reduce bias due to site-specific signatures. |
| Huang (2022) |  |  | x |  | Evaluate the impact of removal of race in eGFR calculation on the accuracy of risk prediction for AKI after PCI | Compared performance (AUC) and calibration of statistical and ML models in Black and in non-Black participants before and after correction for race as a predictor as well as a factor in the eGFR equation | y | Removing the race correction in eGFR calculation had a positive effect on reducing the underestimation of the risk of AKI following PCI for Black patients. Adding race as a covariate improved calibration in both logistic-regression based model and ML (XGBoost) model. |
| Joo (2022) |  |  | x |  | A new eGFR model using the Korean race as a new coefficient was constructed and evaluated. The linear predictor’s race coefficient (coded as “other” race/ethnicity coefficient in the International IgA Nephropathy Prediction Tool) was adjusted using the calibration plot and the Kaplan-Meier plot. | Assessment of calibration using Calibration plots | y | The updated model with the Korean coefficient showed acceptable calibration. |
| Landy (2021) | X |  | x |  | Using new model (LYFS-CT) to predict who would benefit most from broadened screening eligibility criteria Inclusion of sensitive variables Varying cut points or thresholds | Sensitivity for preventable lung cancer deaths and life-years gained | Y | The LYFS-CT model significantly reduced racial disparities, especially for African Americans in screening eligibility and efficiency. |
| Lin (2022) | X |  |  | x | Varying cut points or thresholds (plotted the ceteris paribus cutoff plot of the parity loss for the Black sub-population to determine the optimal confidence threshold for prediction) | Equal opportunity ratio (EOR), predictive parity ratio (PPR), predictive equality ratio (PER), accuracy equality ratio (AER), and statistical parity ratio (SPR) | Y | Adjusting the threshold using this method mitigated the over-prediction of preeclampsia occurrence by the prediction model for Black participants, reducing the predictive equality ratio for Black participants. |
| Mosteiro (2022) |  | x | x |  | Reweighing (pre-processing method) and Prejudice Remover (discrimination-aware regularization [in-processing method]) | Statistical parity, disparate impact ratio, equal opportunity difference, average odds difference | y | -Reweighing improved the fairness metrics. The differences in performance were mostly small, while gains in fairness s were statistically significant at a 95% confidence level. - Prejudice remover improved fairness, though nonsignificantly and at a cost to performance. |
| Noseworthy (2020) |  | x | x |  | Model stratification by race or ethnicity | Performance reevaluation using conventional metrics (AUC, accuracy) | y | The performance was best for the classification of Black/African American and White, non-Hispanic race/ ethnicity. It was less robust for the other subgroups |
| Obermeyer (2019) | x | x |  |  | Adjusting labels in training data; removal of race | Calibration, statistical parity | N/A | Label choice used in algorithm training (e.g. cost of care vs number of chronic conditions) impacted the perception of bias in outcomes. More general labels/outcomes, such as total cost of care, may mask underlying racial disparities, whereas more patient-focused labels/outcomes will reveal them |
| Park (2022) |  | X |  |  | Disparate impact remover (DIR) is a preprocessing algorithm that modifies the feature values of the data set and makes the algorithm discrimination aware at the time of training. | Disparate impact, delta true positive rate, delta false positive rate, statistical parity difference | Y | Fairness was improved, with a slight reduction in accuracy. |
| Park (2021) |  | X |  |  | Reweighing, prejudice remover, and removing race from the models | Disparate impact, equal opportunity difference | Y | There were reduced disparities in prediction of postpartum depression and use mental health services. |
| Perez Alday (2022) |  |  | X |  | Incorporating a bias-penalization term into the loss function of the machine learning model | Model performance between groups | Y | This intervention resulted in similar performance between sexes and races, except for respective differences in female ECGs (higher recording-wise accuracies and lower challenge scores) and Black and Asian subjects (higher and lower recording-wise accuracies, respectively).  Reduced performance was noted in age group 40-49, and recording-wise accuracy decreasing with subsequent decades. |
| Pfohl (2021) |  |  | X | X | Weighting, bias correction, recalibration, varying cut points or thresholds, inclusion of sensitive variables, stratifying models by race | 1) Conditional prediction parity, 2) calibration, and 3) cross-group ranking | Y | The results showed that without fairness-promoting regularization, there were substantial differences in group-level model performance measures such as AUROC, average precision, and cross entropy loss.  Applying conditional regularization penalties will improve fairness but reduce group-level model performance. |
| Pierson (2021) |  | X |  |  | Use of a diverse training set, recalibration? | Difference in prediction accuracy | Y | Models trained under both diverse and non-diverse conditions (i.e., including underrepresented, lower-income, and lower-education groups) outperformed the baseline model. |
| Puyol-Anton (2021) |  |  | X |  | Stratify models (stratified batch sampling), inclusion of sensitive variables (fair meta-learning for segmentation), use of different algorithms (protected group models) | Standard deviation and skewed error ratio of the average .Dice similarity coefficient values | Y | All three bias mitigation strategies (i.e., stratified batch sampling, fair meta-learning for segmentation, and protected group models) were effective in reducing racial bias. |
| Radovanović (2019) |  |  | X | X | Reweighting, varying thresholds | Statistical parity, equality of odds | Y | The reweighting strategy maintained similar predictive performance while improving fairness. The adversarial network showed decreased performance but improved fairness. The equality of odds technique optimized fairness metrics but at the cost of other performance measures. |
| Reeves (2022) |  | X |  |  | Three resampling methods | Equal odds/opportunity | Y | Resampling methods reduced the range in model performance on different racial/ethnic groups by at least 50%. Specifically, equity-focused resampling increased the predictive performance of all four MML models on minoritized racial/ethnic patient groups to approximately match that of the majority (non-Hispanic White) patient group. |
| Segar (2022) | X | X |  |  | Stratify model by race, inclusion of sensitive variable (SDOH) | No explicit fairness metrics but indirect measurement of the effect of incorporating SDOH on fairness. | Y | ML models demonstrated superior performance over the traditional and rederived logistic regressions models using race as a covariate. The addition of SDOH parameters improved the prognostic utility of prediction models in Black patients but not non-Black patients. |
| Segar (2021) | X | X |  |  | Stratify model by race | Model calibration and discrimination | Y | Race-specific and ML-based HF risk models performed better than traditional HF risk and non–race-specific models |
| Thompson (2021) |  |  |  | X | Threshold variation, subgroup recalibration | FNR | N | Although similar features were present between subgroups for predicting opioid misuse, inequities were also present and may only be partially addressed with post-hoc recalibration. |
| Toseef (2022) | X | X |  |  | During algorithm design - transfer learning; "multiethnic experiment schemes" | Possibly demographic parity - comparison of performance between ethnic groups | Y | The results showed that the model improved the transfer learning scheme comparing across ethnic groups with promising AUROC. |
| Weissman (2021) |  |  | X |  | Inclusion of a sensitive variable – ADI | Algorithmic equity/ fairness - is this an example of demographic parity? | N | The inclusion of neighborhood-level data (ADI) may not reliably improve performance or algorithmic equity. |

Abbreviations: ACE, absolute calibration error; ADI, area deprivation index; AKI, acute kidney injury; AUPRC, area under the precision-recall curve; AUROC, area under the receiving operator characteristic curve; eGFR, estimated glomerular filtration rate; eGFR-CKD-EPI, Chronic Kidney Disease Epidemiology Collaboration equation to estimate glomerular filtration rate; eGFR-MDRD, Modification of Diet in Renal Disease equation to estimate glomerular filtration rate; FNR, false negative rate; GS-PPB, Gender Shades Pilot Parliaments Benchmark; HF, heart failure; ICSD, intergroup standard deviation; JFM, joint fairness model; LYFS-CT, Life-Years From Screening with Computed Tomography; ML, machine learning; NLP, natural language processing; PCI, percutaneous coronary intervention; SDOH, social determinant of health; TCE, threshold calibration error; TOLAC, trial of labor after cesarean

**Supplementary Figure 1. Total Publications in Health, Clinical, and Biomedical Sciences Referencing Generative AI
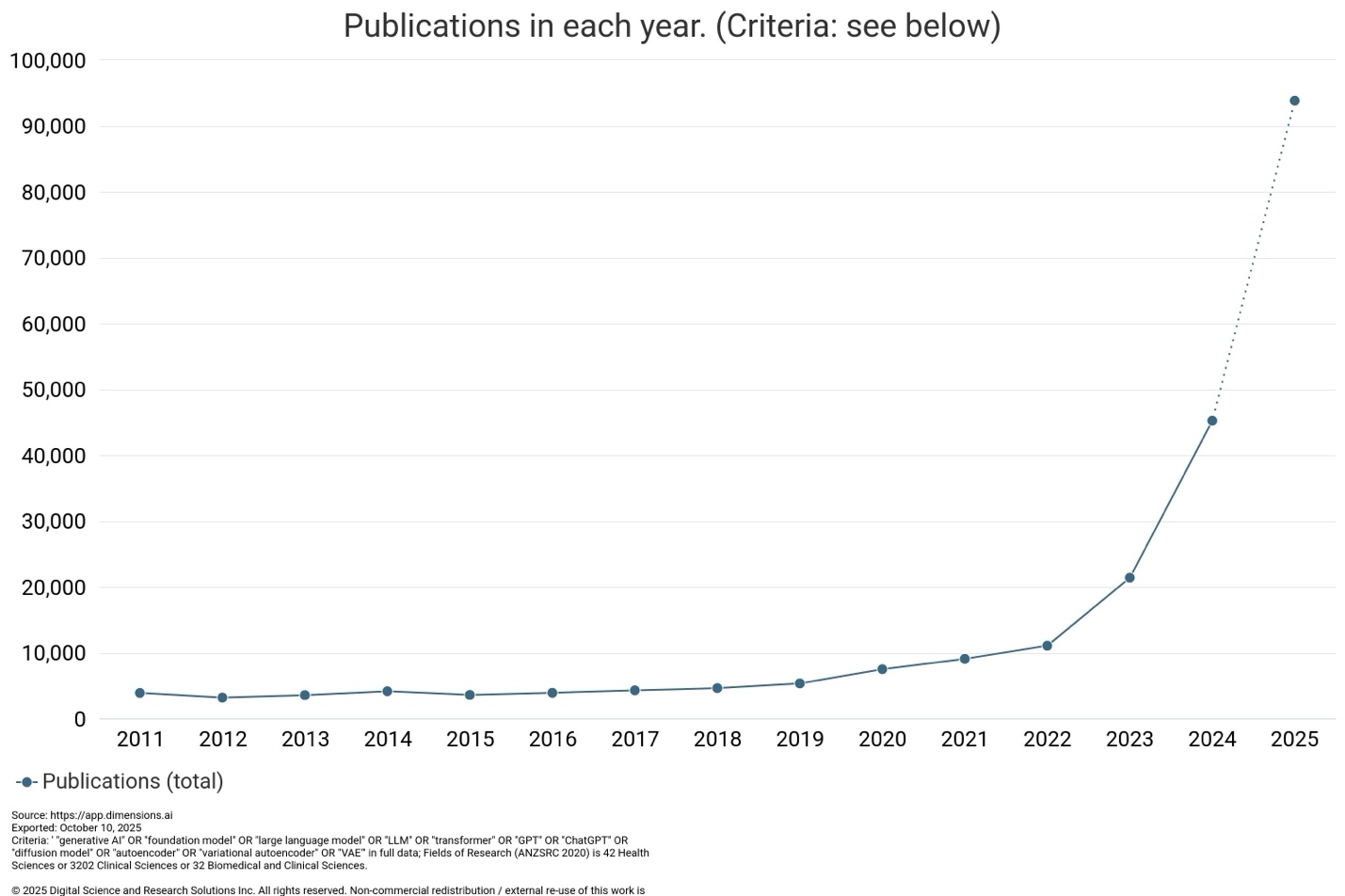
**
